# Molecular and clinical characterization of ICOS expression in breast cancer through large-scale transcriptome data

**DOI:** 10.1371/journal.pone.0293469

**Published:** 2023-12-21

**Authors:** Peng Wang, Qin Zhang, Hengle Zhang, Jianqiang Shao, Hui Zhang, Zunyi Wang

**Affiliations:** 1 Thyroid and Breast Department III, Cangzhou Central Hospital, Cangzhou, Hebei Province, China; 2 Hebei Medical University, Shijiazhuang, Hebei Province, China; Abu Dhabi University, UNITED ARAB EMIRATES

## Abstract

ICOS (Inducible T Cell Costimulator), one of the co-stimulatory B7 superfamily members, was characterized as a co-stimulatory receptor for T-cell enhancement. However, the role of ICOS in breast cancer remains largely unknown. The present study systematically investigated the expression pattern and its relation to clinical characteristics and immunotherapy by integrating multiple clinical cohorts and large-scale gene expression data. This study included 2994 breast tumor samples with transcriptome data and matched clinical data. To make our findings more reliable, we set the TCGA cohort as the discovery set and the METABRIC cohort as the validation set. The expression of ICOS in breast cancer is strongly associated with major clinical and molecular characteristics. There is an association between higher ICOS expression and malignant subtypes and grades of tumors. In addition, gene ontology analysis based on genes significantly correlated with ICOS expression indicated that the expression of ICOS is mainly associated with immune responses and inflammation. We also observed strong correlations between ICOS and other promising immune-checkpoint molecules, including PD1, PDL1, CTLA4, and IDO1. Furthermore, we found that ICOS expression is associated with the response to anti-PDL1 immunotherapy and may serve as a biomarker for immunotherapy prediction. Our results indicated higher ICOS expression is significantly associated with favorable survival in triple-negative breast cancer (TNBC) patients, but not for all subtypes of breast cancer patients. In summary, ICOS correlates with higher malignant breast cancers, and it contributes to the regulation of the immune microenvironment of breast tumors, making it a potential biomarker and immunotherapy target.

## Background

Globally, Cancer-related deaths in women are mostly caused by breast cancer, which is most commonly diagnosed [1, 2]. Despite the advancements in the era of cancer screening and treatments in the past few decades, the treatment for TNBC remains to be the most difficult. The prognosis for patients with TNBC who failed to respond to chemotherapy remains poor.

In recent years, the emergence of cancer immunotherapy and a deeper comprehension of cancer immunity, have provided insights into the treatment of cancer [3, 4]. Accumulating evidence suggests that patients may derive advantages from the inhibition of the suppression of anti-tumor T cell immune responses by blocking the interplay between programmed death-1 (PD1) and ligand-1 (PDL1) [5]. However, immune-checkpoint therapy is not a perfect resolution yet, several limitations and challenges still need to be addressed [6]. Firstly, only a low proportion of patients respond to immunotherapy which might be partially explained by targeting PD1/PDL1 alone might be insufficient to achieve the expected efficacy [6, 7]. For instance, in comparison to monotherapies, Better responses could be achieved by combining antibodies against CTLA-4 and PD-1 [8]. Secondly, a lack of biomarkers to find out the responsive subsets of patients is also a concern with immune-checkpoint therapy [9]. Hence, it is urgent to identify novel potential targets and biomarkers for cancer immune checkpoint therapy.

ICOS, which belongs to the CD28 superfamily that interplays with a ligand ICOSL, acts as a co-stimulatory receptor that activates T-cells in response to a foreign antigen [10, 11]. There are 12% of tumor-infiltrating lymphocytes expressing ICOS in melanoma, making it the most widely expressed costimulatory receptor in the tumor [12]. It has been reported that ICOS is associated with multiple types of tumors in several studies. In gallbladder cancer, it was reported that ICOS could promote the cytotoxic activity of cytokine-induced killer cells by regulating cytokine secretion and cell survival both in vitro and in vivo [13]. In the melanoma tumor microenvironment, ICOS was shown to be expressed on Tregs in tumor-infiltrating cells and was correlated with worse outcomes [14]. ICOS was shown to be involved in enhancing immune tolerance in liver cancer by generating a subset of T-cells with inhibitory function, named T regulatory type 1 cells (Tr1). ICOS/ICOSL signaling has been shown to contribute to anti-tumor immunity through tumor-infiltrating Tr1 cells [15]. However, little data is available on the expression pattern of ICOS and the role of ICOS in breast cancer. Hence, we started this study to systematically look into the molecular and clinical characterization of ICOS in two independent clinical cohorts of breast cancer, as well as its potential to be used as a biomarker for anti-PDL1 immunotherapy.

## Methods

### Patients and samples

We collected transcriptome data and associated clinical data from two independent databases: The Cancer Genome Atlas (TCGA) dataset (https://portal.gdc.cancer.gov/) and the Molecular Taxonomy of Breast Cancer International Consortium (METABRIC) dataset (http://www.cbioportal.org/study/summary). The RNA-seq data were downloaded, filtered, transformed, and normalized using the standard methods implemented in edgeR [16], limma [17], and GDCRNATools packages [18]. Patients lacking complete clinical information were excluded, resulting in a final sample size of 1090 patients from the TCGA cohort [19, 20] and 1994 patients from the METABRIC cohort [20]. Transcriptome data and matched clinical data of the IMvigor210 cohort [20] were accessed and normalized using the IMvigor210 CoreBiologies package in R [20]. We included 298 patients who had received immunotherapy for further analyses.

### Bioinformatics analyses and statistical analysis

An analysis of 32 cancer types from the TCGA database was conducted using the TIMER database to examine the differential expression of ICOS between tumors and adjacent normal tissues. For determining statistical significance, the Wilcoxon test was applied [21]. GOBO database was used to validate the expression pattern of ICOS expression in breast tumor tissues with different molecular subtypes [22]. An analysis of Pearson correlations was performed to identify genes significantly related to ICOS expression. On the basis of the clusterProfiler package, gene ontology (GO) analysis was used to analyze relevant biological functions of ICOS-related genes. A heatmap of ICOS expression and genes associated with immune responses was plotted using the pheatmap package [23]. To calculate immune cell populations based on gene expression measurements the Microenvironment Cell Populations-counter algorithm of R language was used [24]. Survival analysis of ICOS expression was performed by using the Breast Cancer Gene-Expression Miner v4.8 database (bc-GenExMiner v4.8, http://bcgenex.ico.unicancer.fr/) [25]. It is determined whether variables differed between groups using student t-tests, one-way ANOVAs, or Pearson’s Chi-squared tests. In all two-sided tests, statistical significance is defined as p-values less than 0.05.

## Results

### ICOS expression pattern and relationship with molecular and clinical characteristics

**Fig 1:**
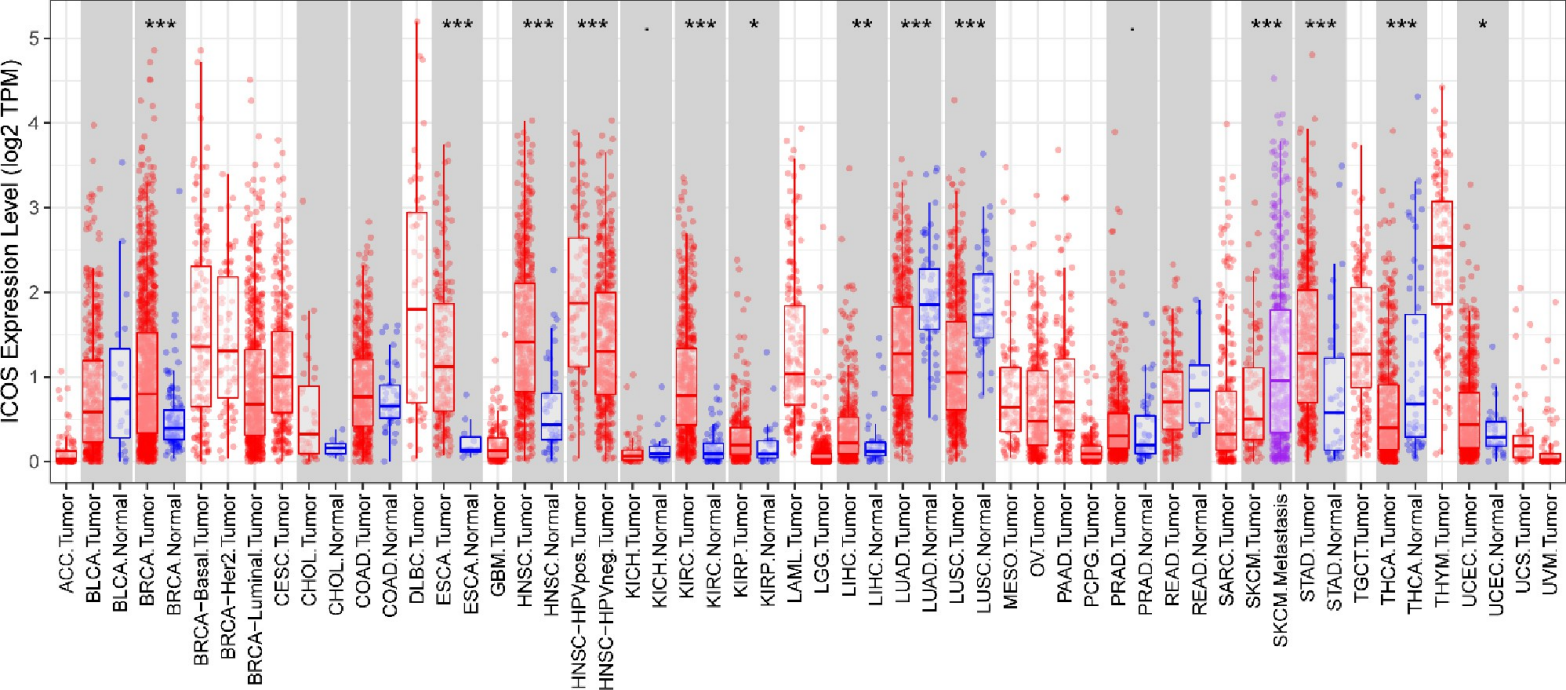
ICOS expression levels in multiple types of human cancers. ICOS expression levels in all tumors and adjacent normal tissues across TCGA (*P < 0.05, **P <0.01, ***P < 0.001).

**Fig 2:**
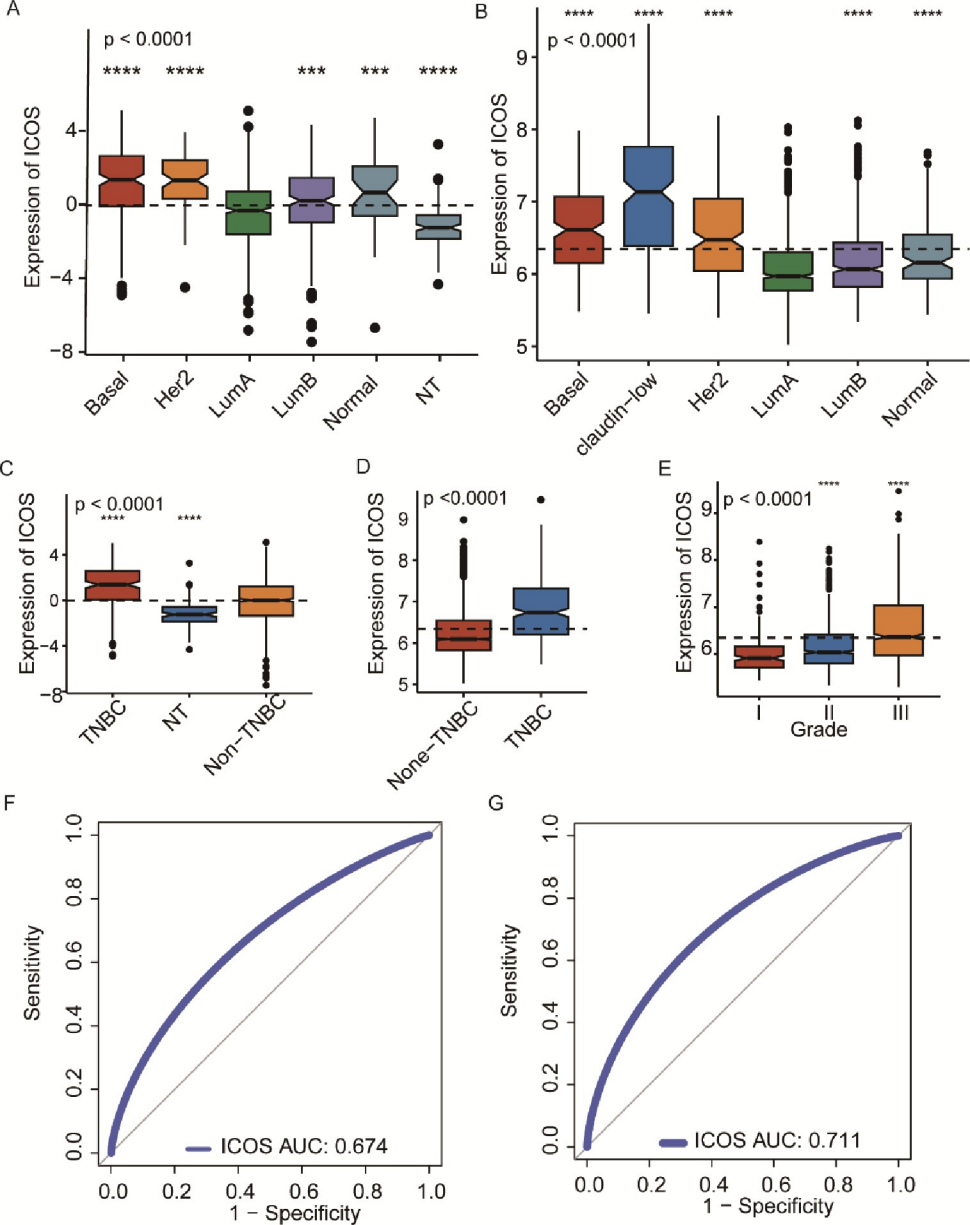
Relationship of ICOS expression and clinical characteristics. ICOS expression pattern in different molecular subtypes and tumor grade in the TCGA (A&C) and METABRIC cohort (B,D&E). (*P < 0.05, **P <0.01, ***P < 0.001, ****P < 0.0001). ROC curve of ICOS expression in the TCGA and METABRIC (F&G).

**Fig 3:**
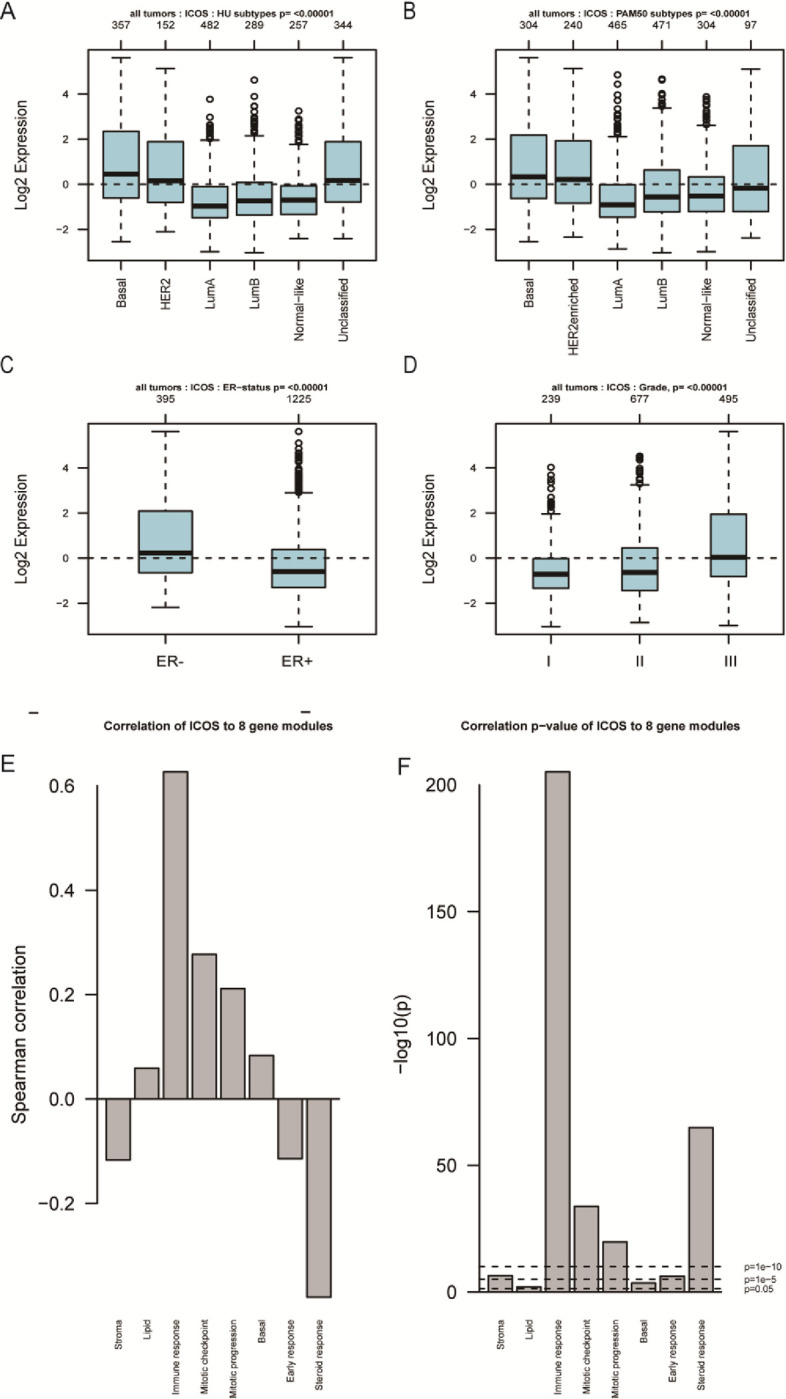
Validation of ICOS expression in independent microarray dataset. ICOS expression pattern in HU subtypes and PAM50 subtypes (A & B). Fig 3C and 3D show the expression pattern of ICOS by ER status and tumor grade. Gene module analyses shows the gene module correlated with ICOS expression (E), and pvalue of each gene module (F).

As shown in **Fig 1**, tumors and adjacent normal tissues express ICOS differently across a variety of cancer types. We found ICOS is significantly overexpressed in the multiple cancers when compared with normal tissues, including BRCA (breast invasive carcinoma), ESCA (esophageal carcinoma), HNSC (head and neck cancer), KIRC (kidney renal clear cell carcinoma), KIRP (kidney renal papillary cell carcinoma), LIHC (liver hepatocellular carcinoma), STAD (stomach adenocarcinoma), UCEC (uterine corpus endometrial carcinoma), however, ICOS is upregulated in LUAD (lung adenocarcinoma), LUSC (lung squamous cell carcinoma), as well as THCA (thyroid carcinoma). To investigate the relationship between ICOS expression and patients’ clinical characteristics, a median cutoff of ICOS expression was used to divide patients of two breast cancer cohorts into low- and high-expression groups. As shown in **Tables 1 and 2**, in both the TCGA and METABRIC cohorts, expression of ICOS is associated with molecular and clinical characteristics including age, tumor stage, estrogen receptor (ER) status, progestogen receptor (PR), HER2 status, and tumor grade. We further investigated the expression pattern of ICOS. As shown in **Fig 2**, ICOS demonstrates significant overexpression in triple-negative breast cancer (TNBC) patients, as well as in the basal-like and HER2+ subtypes in the TCGA cohort (**Fig 2A and 2C**), which is also well validated in the METABRIC cohort (**Fig 2B and 2D**). ICOS expression is significantly upregulated in higher tumor grade in the METABRIC cohort (**Fig 2E**). Additionally, ROC curves are generated for ICOS expression and TNBC subtypes of all breast cancers in order to verify this finding. In the TCGA and METABRIC cohorts, yielded area under the curve (AUC) values of 0.674 and 0.711, respectively (**Fig 2F and 2G**). These findings are further verified in an independent external dataset covering a total of 1881 samples derived from the GOBO database (**Fig 3A–3D**). Through the gene module analysis, we found ICOS expression is strongly associated with the immune response gene module (**Fig 3E and 3F**), which suggests the potential role of ICOS on immune-related functions.

**Table 1:** Association between ICOS mRNA expression and clinicopathologic characteristics in the TCGA cohort.

		Expression		
	Total (N = 1090)	ICOS high (N = 545)	ICOS low (N = 545)	P-value
**Age (years)**				
> = 55	517 (47.4%)	281 (51.6%)	236 (43.3%)	0.008
<55	573 (52.6%)	264 (48.4%)	309 (56.7%)	
**T stage**				
T1	279 (25.6%)	131 (24.0%)	148 (27.2%)	0.018
T2	631 (57.9%)	338 (62.0%)	293 (53.8%)	
T3	137 (12.6%)	63 (11.6%)	74 (13.6%)	
T4	40 (3.7%)	13 (2.4%)	27 (5.0%)	
Unknown	3 (0.3%)	0 (0%)	3 (0.6%)	
**N stage**				
N0	514 (47.2%)	258 (47.3%)	256 (47.0%)	0.839
N1	360 (33.0%)	178 (32.7%)	182 (33.4%)	
N2	120 (11.0%)	65 (11.9%)	55 (10.1%)	
N3	76 (7.0%)	39 (7.2%)	37 (6.8%)	
Unknown	20 (1.8%)	5 (0.9%)	15 (2.8%)	
**M stage**				
M0	907 (83.2%)	452 (82.9%)	455 (83.5%)	0.341
M1	22 (2.0%)	8 (1.5%)	14 (2.6%)	
Unknown	161 (14.8%)	85 (15.6%)	76 (13.9%)	
**AJCC stage**				
I	181 (16.6%)	78 (14.3%)	103 (18.9%)	0.071
II	621 (57.0%)	327 (60.0%)	294 (53.9%)	
III	250 (22.9%)	124 (22.8%)	126 (23.1%)	
IV	20 (1.8%)	7 (1.3%)	13 (2.4%)	
Unknown	18 (1.7%)	9 (1.7%)	9 (1.7%)	
**ER status**				
Negative	236 (21.7%)	170 (31.2%)	66 (12.1%)	<0.001
Positive	803 (73.7%)	355 (65.1%)	448 (82.2%)	
Unknown	51 (4.7%)	20 (3.7%)	31 (5.7%)	
**PR status**				
Negative	343 (31.5%)	213 (39.1%)	130 (23.9%)	<0.001
Positive	694 (63.7%)	312 (57.2%)	382 (70.1%)	
Unknown	53 (4.9%)	20 (3.7%)	33 (6.1%)	
**HER2 status**				
Negative	895 (82.1%)	437 (80.2%)	458 (84.0%)	0.0011
Positive	168 (15.4%)	101 (18.5%)	67 (12.3%)	
Unknown	27 (2.5%)	7 (1.3%)	20 (3.7%)	

**Table 2:** Association between ICOS mRNA expression and clinicopathologic characteristics in the METABRIC cohort.

		Expression		
	Total (N = 1904)	ICOS high (N = 952)	ICOS low (N = 952)	P-value
**Age (years)**				
> = 55	952 (50.0%)	530 (55.7%)	422 (44.3%)	<0.001
<55	952 (50.0%)	422 (44.3%)	530 (55.7%)	
**Tumor size**				
> = 2cm	592 (31.1%)	293 (30.8%)	299 (31.4%)	0.877
<2cm	1292 (67.9%)	646 (67.9%)	646 (67.9%)	
Unknown	20 (1.1%)	13 (1.4%)	7 (0.7%)	
**AJCC stage**				
0	4 (0.2%)	2 (0.2%)	2 (0.2%)	0.179
I	475 (24.9%)	224 (23.5%)	251 (26.4%)	
II	800 (42.0%)	399 (41.9%)	401 (42.1%)	
III	115 (6.0%)	68 (7.1%)	47 (4.9%)	
IV	9 (0.5%)	3 (0.3%)	6 (0.6%)	
Unknown	501 (26.3%)	256 (26.9%)	245 (25.7%)	
**Tumor Grade**				
I	165 (8.7%)	42 (4.4%)	123 (12.9%)	<0.001
II	740 (38.9%)	292 (30.7%)	448 (47.1%)	
III	927 (48.7%)	584 (61.3%)	343 (36.0%)	
Unknown	72 (3.8%)	34 (3.6%)	38 (4.0%)	
**ER status**				
Negative	445 (23.4%)	349 (36.7%)	96 (10.1%)	<0.001
Positive	1459 (76.6%)	603 (63.3%)	856 (89.9%)	
**PR status**				
Negative	895 (47.0%)	577 (60.6%)	318 (33.4%)	<0.001
Positive	1009 (53.0%)	375 (39.4%)	634 (66.6%)	
**HER2 status**				
Negative	1668 (87.6%)	782 (82.1%)	886 (93.1%)	<0.001
Positive	236 (12.4%)	170 (17.9%)	66 (6.9%)	

### ICOS-related biological process

**Fig 4:**
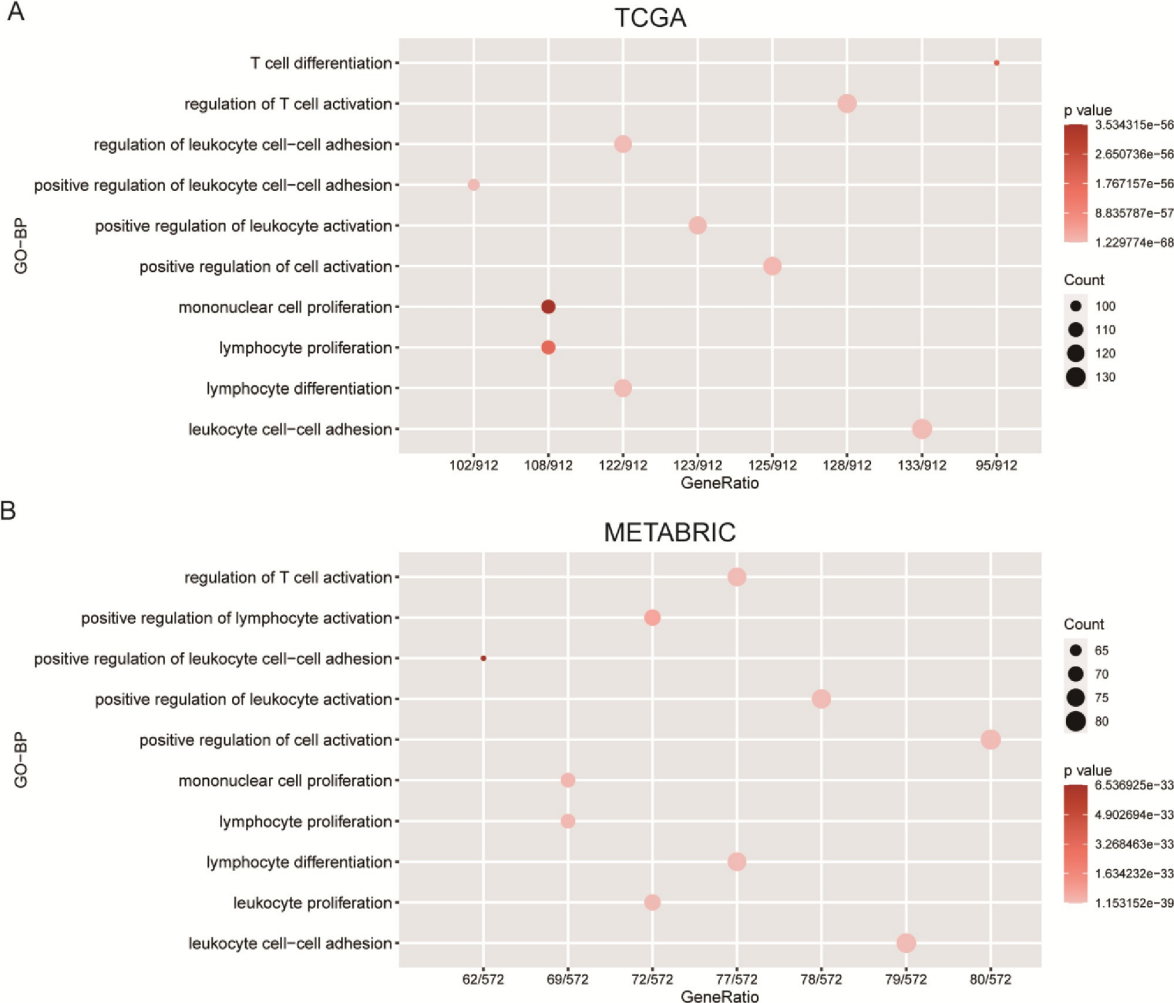
ICOS was closely correlated with immune functions in breast cancer. Gene ontology analysis showed that ICOS is mainly involved in immune response and inflammatory response in both the TCGA and METABRIC databases **(A and B**).

Genes significantly correlated with ICOS expression (|R| > 0.4 & P<0.05) are screened out in the TCGA (988 genes) and METABRIC (660 genes) cohorts, and the detailed list of these genes are provided in **S1 and S2 Tables**. These genes were further analyzed by functional annotation enrichment using clusterProfile package in R. In both the TCGA and METABRIC cohorts, we found that the majority of these genes are enriched in inflammatory and immune-related biological processes including regulation of T cell activation, positive regulation of leukocyte activation, positive regulation of cell activation, lymphocyte proliferation, lymphocyte differentiation, leukocyte cell−cell adhesion (**Fig 4A and 4B**). These results are consistent with the gene module analyses from the 1881-sample dataset.

### ICOS-related immune response

**Fig 5:**
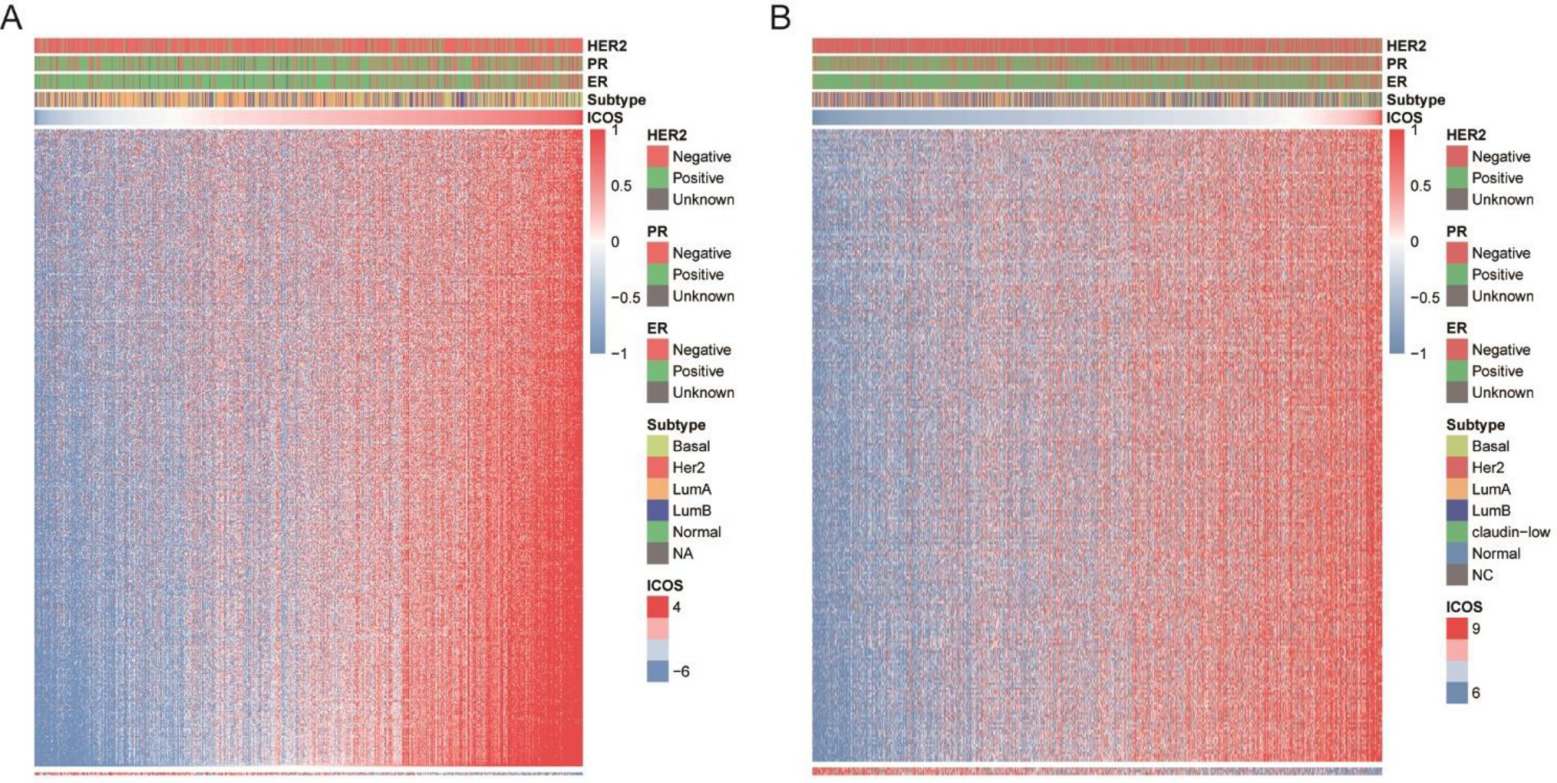
ICOS-related immune responses. The majority of immune-related genes are positively correlated with ICOS expression in TCGA and METABRIC databases, while a small number of genes are negatively associated **(A and B)**.

The Immunology Database and Analysis Portal (ImmPort) database was used to collect certain immune-related genes to further examine the role of ICOS in immune response (n = 4723). We present a heatmap of these immune-related genes most relevant to ICOS expression (Pearson, |R|>0.4&P<0.05). Among these selected genes, we found that ICOS is positively correlated with 504 and 257immune-related genes in the TCGA and METABRIC cohorts, respectively, while only two and three immune-related genes are negatively correlated with ICOS, respectively (**Fig 5A and 5B**). Therefore, ICOS expression is positively associated with most relevant immune responses, but negatively correlated with few relevant immune responses in both the TCGA and METABRIC cohorts.

### Association of ICOS expression and immune cell populations

**Fig 6:**
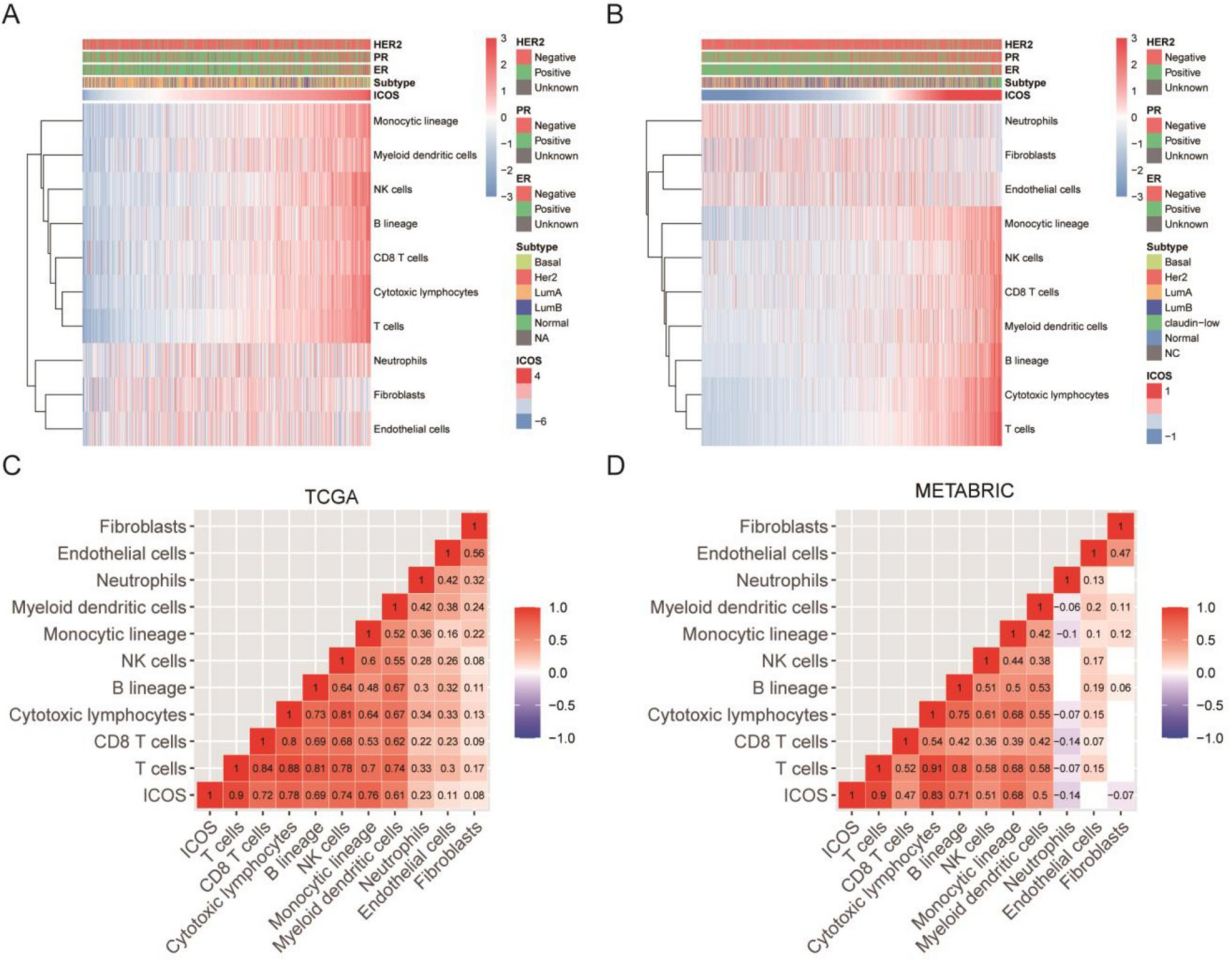
Relationship between ICOS expression and immune cell populations in the TCGA and METABRIC databases. (A-D). The color bar denotes the value of normalized expression of ICOS.

We further clarified the association of ICOS expression and immune cell populations. By relying on transcriptome data, we calculate the absolute abundance of immune cell populations in each sample using the Microenvironment Cell Populations-counter method developed by Etienne Becht et al [24]. The immune cell population abundance profile correlated with ICOS expression is shown with a heatmap (**Fig 6A and 6B**). We found that ICOS expression is strongly associated with most immune cell populations in both the TCGA and METABRIC cohorts, but the correlation between ICOS expression and the abundance of the stromal cell population is not consistent in these two datasets (**Fig 6C and 6D**). Particularly, the strongest correlation is observed between ICOS expression and T cell (R = 0.9), this result is well mutually validated in both the TCGA and METABRIC datasets. These results suggest the important role of ICOS expression and T-cell immunity.

### Association of ICOS and immune-checkpoint molecules

**Fig 7:**
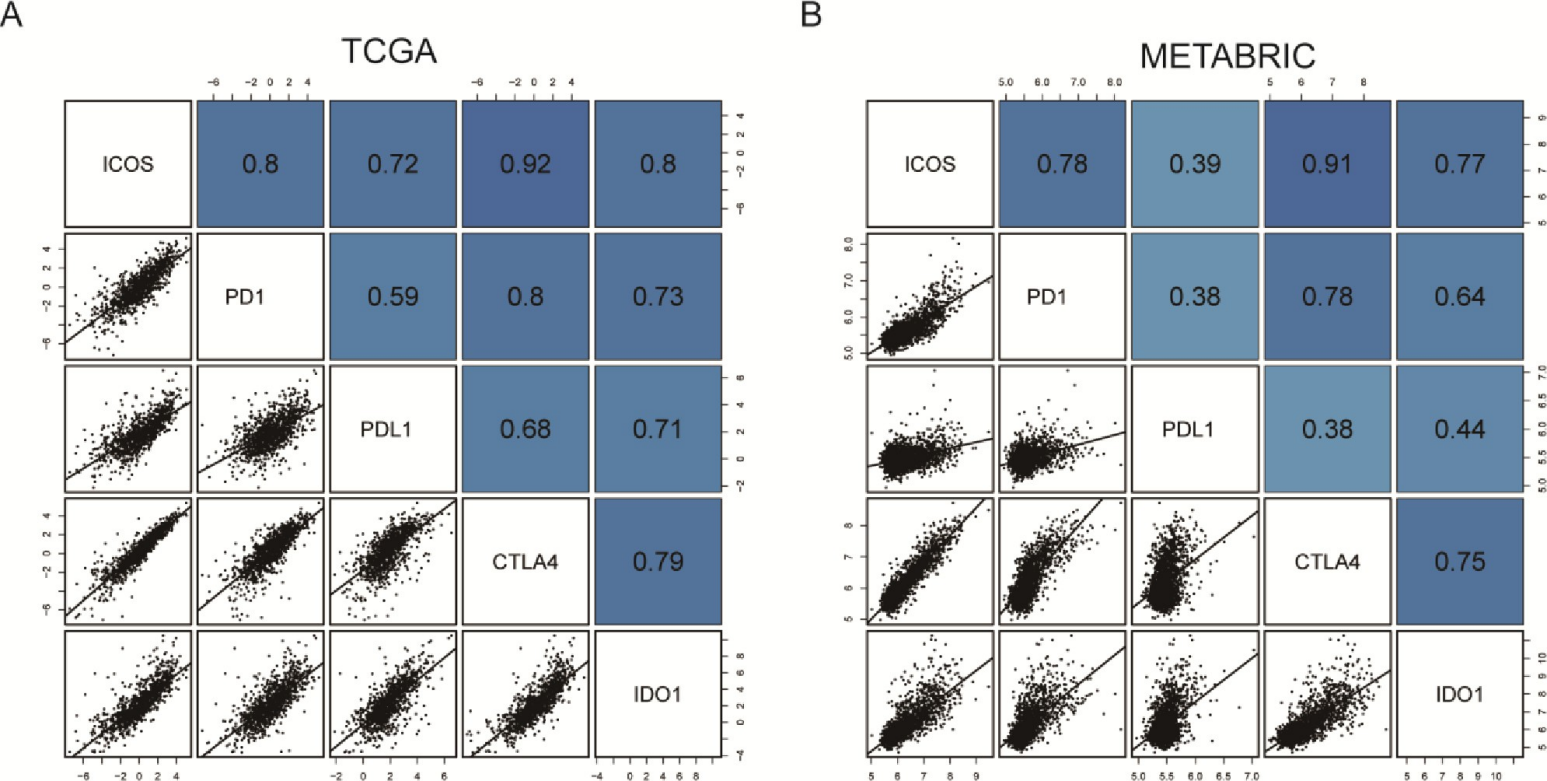
ICOS expression is strongly correlated with immune checkpoint molecules in the TCGA and METABRIC databases. (A-B). The shade of color denotes the correlation coefficient of ICOS expression and other molecules.

Accumulating evidence suggests that combination therapy with inhibition of immune-checkpoint molecules contributes to improved clinical benefit in both preclinical and clinical studies [9]. To further investigate the potential association between ICOS and other important immune-checkpoint molecules, we also included the additional genes of immune-checkpoint s. In the discovery TCGA dataset, we observed that ICOS expression is strongly correlated with important immune-checkpoint members, including PD1, PDL1, CTLA4, and IDO1 (**Fig 7A**). In the METABRIC dataset, these results are well validated (**Fig 7B**).

### Association of ICOS expression and anti-PD-L1 response

**Fig 8:**
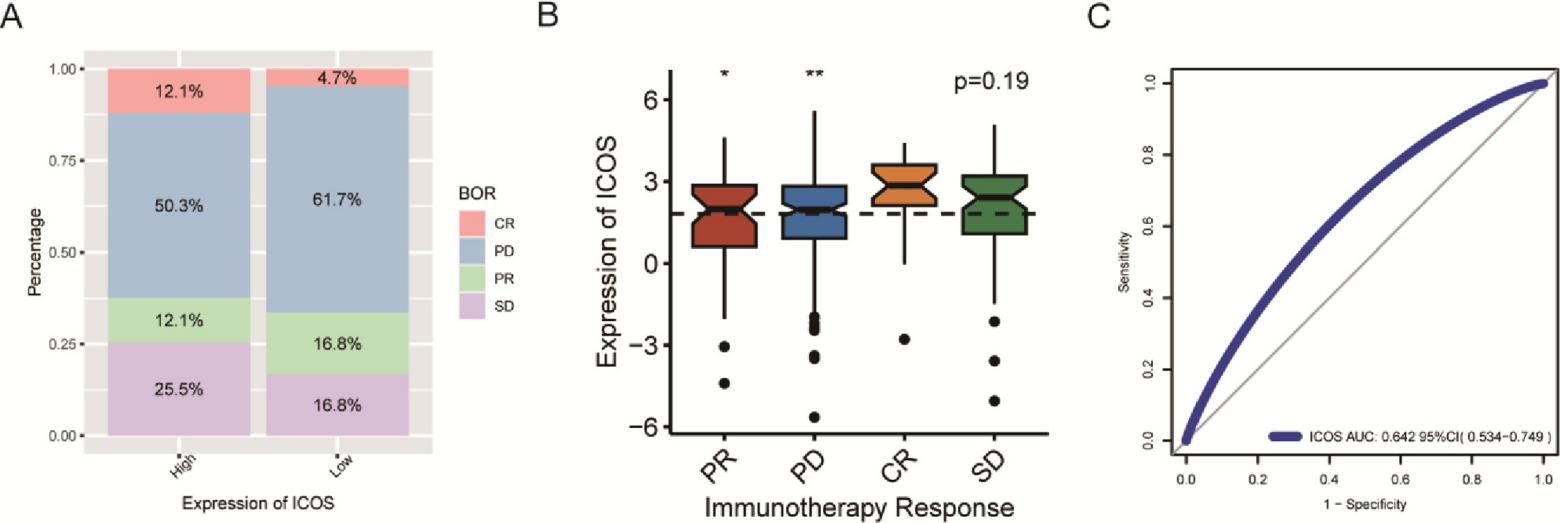
ICOS as a biomarker for anti-PD-L1 response. Distribution pattern of patients accepting anti-PD-L1 response by high- and low-expression group of ICOS (A). Expression pattern of ICOS in different groups of anti-PD-L1 response (B). ROC curve of ICOS expression for predicting CR. Progressive disease (PD)/stable disease (SD); R represents complete response (CR)/partial response (PR). (*P < 0.05, **P <0.01, ***P < 0.001, ****P < 0.0001).

To assess whether ICOS expression could predict responses of patients to immune-checkpoint inhibitors. A total of 298 patients from the IMvigor210 cohort who received anti-PD-L1 treatment were included in our study. Patients were divided into low- and high-expression groups according to the median cutoff of ICOS expression. We found that patients with higher expression of ICOS experienced greater rate of complete response (CR) (**Fig 8A**). As shown in **Fig 8B**, ICOS is found to be significantly overexpressed among patients with CR when compared with patients with progressive disease (PD) or partial response (PR). ROC curve for ICOS expression and patients with response of CR was performed, and AUC reached 0.642 (**Fig 8C**). These findings suggest the ICOS expression is associated with the response of patients accepting immunotherapy and could be considered as a marker for response prediction.

### ICOS expression is correlated with survival of TNBC patients

**Fig 9:**
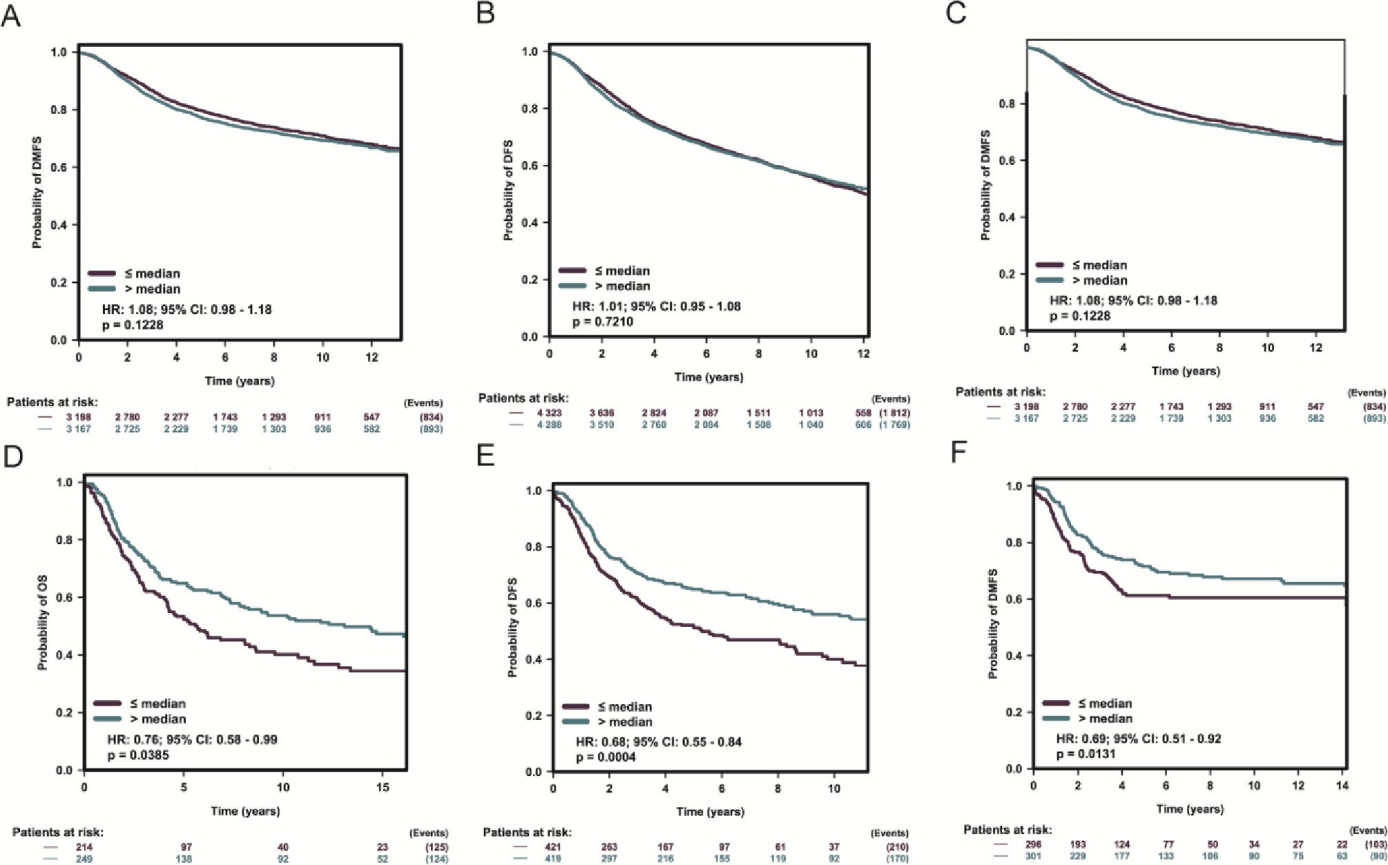
ICOS expression and survival of breast cancer patients. Association of ICOS expression and OS, DFS, DMFS in the entire breast cancer cohort (A-C) and TNBC cohort (D-F).

To explore the prognostic role of ICOS for breast cancer patients, we further analyzed the expression of ICOS and survival in a microarray dataset including a total of 8069 patients derived from the bc-GenExMiner v4.4 database [25]. For all breast cancer patients as a full cohort, we found no significant association between ICOS expression and survival of breast cancer patients (**Fig 9A–9C**), including disease free survival (DFS), overall survival (OS), and distant metastasis free survival (DMFS). As we have found that ICOS expression is more likely to be overexpressed in more malignant breast cancer subtypes, we further investigate the prognostic role of ICOS in TNBC patients. Interestingly, we found higher ICOS expression is associated with improved OS, DFS as well as DMFS (**Fig 9D–9F**).

## Discussion

Our data indicated that ICOS expression is correlated with the degree of malignancy of breast tumor and the survival of patients with TNBC. Wang et al. [26] analyzed the role of ICOS in gliomas, they supported the idea that ICOS expression is associated with mesenchymal subtype of gliomas. Inconsistently, the authors found that ICOS expression is positively correlated with immune score and stroma score, and associated with a worse prognosis. As we found that the ICOS expression is associated with a better prognosis for breast cancer patients, and positively correlated with immune cell sores, especially T-cell immune responses, but not for stromal cells, which suggests the varied role of ICOS expression in different tumors. This phenomenon might be explained by the heterogeneity of cancers and the fact that the immune regulatory pattern of ICOS might be vary in different tumors.

Currently, the essential strategy for cancer immunotherapy aims to block the coinhibitory receptors, such as PD1 and CTLA4, which is no doubt an important advance for cancer treatment. As for the low rate of response to immunotherapy, it would be helpful to seek out effective and novel biomarkers to predict the efficacy of immunotherapy and identify suitable subsets of populations. In preclinical studies, activating ICOS by an agonist is not sufficient to mediate anti-tumor effect, combining blocking inhibitory checkpoints and ICOS agonist mAbs should be considered as a promising strategy for improving efficacy for cancer immunotherapy. However, increasing evidence suggests the key role of ICOS in anti-tumor response induced by anti-CTLA4 therapy, such as ipilimumab or tremelimumab. For instance, increased expression of ICOS was observed on CD4+ T cells from peripheral blood and tumor tissues of patients receiving anti-CTLA4 therapy in several types of cancer, including breast cancer, lung cancer, and bladder cancer [27–29]. With a preclinical model using prostate cancer and mouse melanoma, Fan et al. provided evidence that combining ICOS engagement and CTLA4 blockage could produce synergistic anti-tumor effect [30]. Using tumor cells transduced with ICOSL as a vaccine, the authors observed improved effects of antitumor T effector cells, which is reflected by a higher ratio of intratumoral CD8/Treg and CD4+ Teff/Treg cell.

ICOS displays a dualistic in tumors including both anti-tumor effect by promoting T-cell response and enhancing tumor progression. Targeting ICOS and CTLA4 together was demonstrated to promote the anti-tumor effect, and the expression of ICOS could serve as a biomarker for predicting the efficacy of anti-CTLA4 therapy. However, the role of ICOS expression for anti-PD1 and anti-PDL1 therapy is yet to be illustrated.

ICOS was shown to be involved in the regulation of the tumor immunity through two different perspectives. One the one hand, ICOS could act as a promoter of antitumor effect by activating the antitumor cytotoxic T cells. On the other hand, ICOS could contribute to an-tumor immunity for the regulation of function and maintenance of Tregs. Due to the dual effect of ICOS, we hypothesize it could be a useful predictive biomarker for patients’ responses to immunotherapy. ICOS has been shown to be expressed by PD-1+CD8+ T-cells after anti-PD1 therapy in lung cancer. These clues suggest that ICSO might be a promising biomarker for the emerging cancer immunotherapy [31]. In this study, our data support this idea by using the imvgor210 cohort data, we found ICOS expression was associated the rate of CR of patients accepting anti-PDL1 therapy.

As for breast cancer, ICOS gene polymorphisms were reported to be associated with the risk and characteristics of breast cancer in a Chinese patient cohort [32]. Retrospective cohort data reported that the overexpression of ICOSL protein was associated with worse outcomes in breast cancer patients [33]. ICOS expression was found on tumor infiltrating and proliferative Tregs, and was suggested to be associated with poor prognosis in a cohort of 120 patients with invasive nonmetastatic breast tumors [34]. This is inconsistent with our studies, this might be due to the small sample size of previous studies. Our results are based on a large sample size of patients, and validated in independent datasets, which could be more reliable. As we currently know, ICOS is positive regulator of T-cell function in anti-tumor immune response, but also involving the function of immune escape as its association with suppressive Tregs. Thus, whether should we use an antagonist or an agonist mAb remains to be known. As it has been well established, breast cancer is the so-called “cold tumor” that with the paucity of tumor T-cell infiltration, we suggest ICOS agonist mAbs might be of promising to promote the anti-tumor T-cell responses. As far as we know, this is the largest and most comprehensive study for depicting the molecular and clinical features of ICOS in breast cancer. However, findings of the current study should be considered in the context of limitations. While bioinformatics analysis can generate hypotheses and predictions, experimental validation is crucial to confirming the biological significance of the findings in future study. Bioinformatics analysis alone might not be sufficient to directly inform therapeutic strategies without further experimental and clinical investigations.

## Supporting information

S1 Table Genes significantly correlated with ICOS expression in the TCGA cohort. (PDF)

S2 Table Genes significantly correlated with ICOS expression in the METABRIC cohort. (PDF)

S1 Checklist (DOCX)
