# Supplementary material for: Molecular and clinical characterization of ICOS expression in breast cancer through large-scale transcriptome data
Source: PLoS One. 2023 Dec 21;18(12):e0293469. doi: 10.1371/journal.pone.0293469 (PMC10734928; doi:10.1371/journal.pone.0293469)
Supplement: S1 Table — (PDF) [file pone.0293469.s001.pdf]

Table S1. Genes significantly correlated with ICOS expression in the TCGA cohort

	symbol	correlation	pvalue
1	ICOS	1	0
2	TIGIT	0.939413	0
3	CTLA4	0.935441	0
4	GBP5	0.915441	0
5	SIRPG	0.901544	0
6	CD2	0.899142	0
7	LTA	0.885765	0
8	IL2RG	0.88271	0
9	SLA2	0.879016	0
10	ZBED2	0.878638	0
11	SNX20	0.878441	0
12	CXCR6	0.875546	0
13	SP140	0.87445	0
14	CD96	0.873237	0
15	SH2D1A	0.871022	0
16	ITK	0.868933	0
17	FOXP3	0.868135	0
18	CD3G	0.868047	0
19	CCR5	0.866336	0
20	CXCL9	0.864772	0
21	SLAMF1	0.864401	0
22	PTPN7	0.862035	0
23	CD5	0.859063	0
24	SLAMF6	0.856644	0
25	CD3D	0.85385	0
26	PYHIN1	0.853585	0
27	LCK	0.853466	0
28	TNFRSF9	0.85329	0
29	CD3E	0.852883	0
30	CXCR3	0.852684	0
31	IL2RA	0.8513	0
32	IL12RB1	0.849774	0
33	CD247	0.848156	0
34	CD38	0.846996	0
35	UBASH3A	0.845389	0
36	BTLA	0.840588	0
37	TRAT1	0.840219	0
38	TBX21	0.83974	0
39	IDO1	0.837882	0
40	ZNF831	0.837019	0
41	SLFN12L	0.835938	0
42	SLAMF7	0.834184	0
43	AIM2	0.833673	0
44	CXCL10	0.832198	0
45	SIT1	0.830718	0
46	CXCL11	0.830358	0
47	TIFAB	0.829663	0
48	CCL5	0.829598	0
49	FCRL3	0.825551	0
50	CD48	0.822174	0
51	IKZF1	0.821027	0

52	PTPRC	0.820517	0
53	SAMD3	0.820184	0
54	IFNG	0.819835	0
55	FASLG	0.819301	0
56	GZMB	0.81895	0
57	PLEK	0.818162	0
58	GPR171	0.818084	0
59	CD226	0.81515	0
60	SPOCK2	0.813091	0
61	IL2RB	0.81162	0
62	LCP2	0.810688	0
63	PRF1	0.810196	0
64	PDCD1	0.810183	0
65	LY9	0.808721	0
66	CCR8	0.80762	0
67	GBP1	0.807231	0
68	SASH3	0.805503	0
69	IL21R	0.804383	0
70	GZMA	0.804223	0
71	CD27	0.803977	0
72	LAMP3	0.80395	0
73	CD53	0.802592	0
74	IRF4	0.801763	0
75	BIN2	0.80149	0
76	PRKCB	0.800914	0
77	PLA2G2D	0.799872	0
78	CD6	0.799739	0
79	GBP4	0.799445	0
80	GPR174	0.798672	0
81	PARP15	0.798382	0
82	C16orf54	0.796945	0
83	CRTAM	0.796927	0
84	SLAMF8	0.79316	0
85	IL18RAP	0.792933	0
86	FYB1	0.792857	0
87	PDCD1LG2	0.792828	0
88	TRAF3IP3	0.792294	0
89	IL10RA	0.791632	0
90	NLRC5	0.790867	0
91	GFI1	0.789853	0
92	CCR4	0.789326	0
93	NKG7	0.789216	0
94	ICAM3	0.788311	0
95	EOMES	0.788237	0
96	STAT4	0.788078	0
97	KLHL6	0.786458	0
98	CD8A	0.786116	0
99	SPN	0.785725	0
100	CD7	0.783858	0
101	LAX1	0.783236	0
102	THEMIS	0.781472	0
103	ZC3H12D	0.781112	0
104	ZAP70	0.780606	0

105	PTPN22	0.780464	0
106	EPSTI1	0.780011	0
107	TNFSF13B	0.778862	0
108	IL12B	0.778317	0
109	SCML4	0.777465	0
110	CST7	0.777391	0
111	TNIP3	0.776165	0
112	ARHGAP25	0.775909	0
113	CCR7	0.774653	0
114	JAK3	0.774375	0
115	PRKCQ	0.773315	0
116	CCR2	0.772637	0
117	ZBP1	0.772135	0
118	TESPA1	0.770677	0
119	GZMK	0.769845	0
120	IL18BP	0.768778	0
121	TNFSF14	0.768626	0
122	TAGAP	0.76635	0
123	IKZF3	0.763773	0
124	SAMSN1	0.76375	0
125	MAP4K1	0.761839	0
126	CD52	0.757411	0
127	SSTR3	0.756738	0
128	TFEC	0.755531	0
129	DOCK2	0.75553	0
130	CD40LG	0.7547	0
131	CLNK	0.754694	0
132	IRF8	0.754424	0
133	LAG3	0.753507	0
134	EVI2B	0.752557	0
135	P2RY10	0.751651	0
136	ARHGAP9	0.750249	0
137	IL7R	0.749435	0
138	GNLY	0.748825	0
139	NCKAP1L	0.748305	0
140	LYZ	0.748088	0
141	CD4	0.747898	0
142	LPXN	0.747232	0
143	ARHGAP30	0.746721	0
144	ADAMDEC1	0.746616	0
145	LILRB2	0.744921	0
146	CD274	0.743797	0
147	SELL	0.74342	0
148	TMC8	0.743342	0
149	NCR3	0.742909	0
150	APOBEC3H	0.741842	0
151	S1PR4	0.740455	0
152	ZNF683	0.738059	0
153	CSF2RB	0.737478	0
154	XCL2	0.737131	0
155	SAMHD1	0.736704	0
156	XCL1	0.736156	0
157	BCL2A1	0.735263	0

158	TTC24	0.735072	0
159	ITGB7	0.734407	0
160	PATL2	0.733118	0
161	FAM78A	0.732877	0
162	DOK2	0.732444	0
163	HLA-DMB	0.731944	0
164	CIITA	0.73183	0
165	FGD2	0.731606	0
166	CD86	0.730945	0
167	GPR18	0.730564	0
168	KCNA3	0.730463	0
169	TLR8	0.729663	0
170	CTSS	0.729634	0
171	CALHM6	0.728256	0
172	HLA-DQA1	0.728203	0
173	AOAH	0.727971	0
174	CD37	0.727955	0
175	SRGN	0.727697	0
176	HLA-DRA	0.725406	0
177	CD28	0.724516	0
178	CTSW	0.722633	0
179	OR2I1P	0.722463	0
180	LILRB1	0.721885	0
181	GRAP2	0.720695	0
182	PSTPIP1	0.720253	0
183	SCIMP	0.719907	0
184	ZNF80	0.719791	0
185	CYTIP	0.719297	0
186	SPIB	0.717943	0
187	TOX	0.717676	0
188	CD8B	0.717278	0
189	CCL4	0.717044	0
190	TNFRSF1B	0.716287	0
191	BTK	0.715481	0
192	ARHGAP15	0.714812	0
193	JAML	0.713668	0
194	CD244	0.712871	0
195	STAP1	0.712789	0
196	TNFRSF8	0.712692	0
197	KLRD1	0.711481	0
198	CARD11	0.71109	0
199	LAIR2	0.710498	0
200	CD80	0.710425	0
201	SH2D2A	0.709652	0
202	TCL1A	0.708589	0
203	UBD	0.707606	0
204	WAS	0.705404	0
205	SLA	0.704921	0
206	APOBEC3G	0.704801	0
207	CARD17	0.70443	0
208	P2RY8	0.70387	0
209	CXorf21	0.703806	0
210	CLEC6A	0.703638	0

211	FPR3	0.703453	0
212	C11orf21	0.703268	0
213	WDFY4	0.702964	0
214	TAP1	0.702948	0
215	KIR2DL4	0.702817	0
216	PSMB9	0.702464	0
217	CD200R1	0.701954	0
218	NCF1	0.701858	0
219	IDO2	0.701389	0
220	NCR1	0.7007	0
221	TNFAIP3	0.700338	0
222	MEI1	0.699975	0
223	FERMT3	0.698097	0
224	CLEC4C	0.697314	0
225	IL10	0.697285	0
226	PLAC8	0.696816	0
227	GTSF1L	0.696742	0
228	CD300LF	0.696599	0
229	NUGGC	0.696521	0
230	XCR1	0.695853	0
231	CD72	0.694887	0
232	BCL11B	0.69475	0
233	MYO1G	0.694057	0
234	FMNL1	0.693559	0
235	HLA-DOB	0.693319	0
236	TMEM150I	0.693144	0
237	LAIR1	0.692595	0
238	HCLS1	0.692494	0
239	GZMH	0.691992	0
240	STAT1	0.691948	0
241	CD69	0.691635	0
242	CLEC7A	0.69152	0
243	IL4I1	0.690935	0
244	CCL13	0.690427	0
245	RUNX3	0.690063	0
246	MS4A1	0.689998	0
247	PIK3CD	0.689702	0
248	ACAP1	0.689605	0
249	MPEG1	0.688974	0
250	PIK3AP1	0.688548	0
251	SERPINB9	0.686523	0
252	PIK3R5	0.686152	0
253	CORO1A	0.686125	0
254	NLRC3	0.685148	0
255	FCRL5	0.68385	0
256	BIRC3	0.683312	0
257	LTB	0.682725	0
258	FGL2	0.681103	0
259	RASSF4	0.680322	0
260	CYBB	0.679928	0
261	LILRB4	0.679866	0
262	IRF1	0.679108	0
263	TAP2	0.679072	0

264	RASAL3	0.678518	0
265	TRIM22	0.678466	0
266	CCL8	0.677868	0
267	CD19	0.677656	0
268	B2M	0.676917	0
269	WNT10A	0.676401	0
270	SLC7A7	0.676297	0
271	IPCEF1	0.676235	0
272	FCRLA	0.675753	0
273	MAL	0.675682	0
274	GPR183	0.675343	0
275	TRAF1	0.675259	0
276	FCRL6	0.674416	0
277	FUT7	0.674266	0
278	TBC1D10C	0.673736	0
279	WIPF1	0.67345	0
280	CR1L	0.672932	0
281	TREML2	0.671888	0
282	MNDA	0.671864	0
283	GAB3	0.671499	0
284	PTAFR	0.671467	0
285	CD79A	0.671412	0
286	MS4A6A	0.671098	0
287	CD74	0.669367	0
288	HLA-E	0.668942	0
289	GZMM	0.667824	0
290	GPR65	0.667705	0
291	AKNA	0.667013	0
292	CXCL13	0.666467	0
293	FCRL2	0.665567	0
294	NCF4	0.665186	0
295	KCNJ10	0.664906	0
296	THEMIS2	0.664823	0
297	HLA-DMA	0.664685	0
298	IL15RA	0.664483	0
299	CLEC4A	0.664407	0
300	POU2AF1	0.663433	0
301	HLA-DOA	0.663024	0
302	SLCO5A1	0.661706	0
303	LAPTM5	0.661013	0
304	GIMAP4	0.660765	0
305	KLRB1	0.658882	0
306	STX11	0.658707	0
307	CTSC	0.65825	0
308	GNGT2	0.657121	0
309	CCR1	0.656621	0
310	CLEC12A	0.656155	0
311	ASB2	0.655725	0
312	VNN2	0.655668	0
313	IL18R1	0.654737	0
314	ITGB2	0.654301	0
315	CD84	0.654054	0
316	BTN3A1	0.653384	0

317	IL15	0.652295	0
318	SLC12A3	0.651884	0
319	HLA-DRB1	0.651728	0
320	FGR	0.651616	0
321	ITGAL	0.651176	0
322	DTHD1	0.651121	0
323	TRPV2	0.650856	0
324	IL21	0.650802	0
325	APOBEC3A	0.650221	0
326	PARVG	0.649917	0
327	CD40	0.649329	0
328	C1QB	0.648842	0
329	SIGLEC10	0.648815	0
330	BTN3A3	0.648421	0
331	APBB1IP	0.648319	0
332	SELPLG	0.648288	0
333	KLRC1	0.647733	0
334	LGALS2	0.64662	0
335	HLA-DPA1	0.646486	0
336	F5	0.646214	0
337	IGSF6	0.645702	0
338	CASP1	0.64552	0
339	GIMAP5	0.644785	0
340	CLEC4E	0.644668	0
341	XIRP1	0.644656	0
342	IL16	0.644616	0
343	PIM2	0.644462	0
344	MYO1F	0.64427	0
345	INPP5D	0.643711	0
346	TNFAIP8	0.64355	0
347	LILRB3	0.643256	0
348	SOCS1	0.643244	0
349	WARS	0.642607	0
350	KCNAB2	0.642442	0
351	CYTH4	0.64238	0
352	FCRL1	0.641625	0
353	TRABD2A	0.641399	0
354	CXCR5	0.641323	0
355	CD83	0.64031	0
356	VCAM1	0.639383	0
357	IL12RB2	0.638094	0
358	FCN1	0.637734	0
359	CNR2	0.636693	0
360	NCF2	0.63629	0
361	C1orf162	0.636217	0
362	POU2F2	0.634929	0
363	CARD16	0.634399	0
364	KBTBD8	0.634028	0
365	ADA2	0.633936	0
366	FPR2	0.633639	0
367	ABCD2	0.632851	0
368	APOL1	0.632596	0
369	KIR3DL2	0.631745	0

370	C1QA	0.631622	0
371	RCSD1	0.631424	0
372	HLA-DPB1	0.631242	0
373	APOL6	0.630778	0
374	TLDC2	0.629871	0
375	MS4A4A	0.629399	0
376	BANK1	0.628307	0
377	SLC15A3	0.628085	0
378	HLA-F	0.62703	0
379	LYN	0.626933	0
380	MCOLN2	0.626666	0
381	LILRA5	0.626642	0
382	C1QC	0.626581	0
383	PLA2G7	0.626082	0
384	LCP1	0.625651	0
385	TMIGD2	0.625312	0
386	HCK	0.624469	0
387	CLEC4D	0.623712	0
388	IL9R	0.623667	0
389	CD180	0.623599	0
390	EBI3	0.623354	0
391	HAVCR2	0.623149	0
392	PLCB2	0.622949	0
393	KLRK1	0.621759	0
394	IL32	0.621011	0
395	ADGRG5	0.620914	0
396	HCST	0.62072	0
397	TNFSF8	0.620578	0
398	CXorf65	0.620439	0
399	VAV1	0.619113	0
400	ADGRE5	0.618894	0
401	HTRA4	0.61737	0
402	ETV7	0.617341	0
403	RGS18	0.617042	0
404	CLIC2	0.616029	0
405	HLA-B	0.614943	0
406	RAB33A	0.614501	0
407	SAMD9L	0.614454	0
408	LRMP	0.614013	0
409	PAX5	0.613415	0
410	RIPOR2	0.613138	0
411	GMFG	0.612705	0
412	ITGAX	0.612543	0
413	TNFAIP8L2	0.6123	0
414	PTGER4	0.612156	0
415	HLA-DQB1	0.611025	0
416	EVI2A	0.609099	0
417	C15orf53	0.608677	0
418	LIMD2	0.607782	0
419	KIF21B	0.607504	0
420	PNOC	0.606917	0
421	P2RY6	0.606231	0
422	BTN3A2	0.606184	0

423	MLKL	0.60535	0
424	GPR55	0.605048	0
425	BEND4	0.60475	0
426	SEMA4D	0.604122	0
427	BLK	0.604	0
428	PIK3CG	0.603265	0
429	SEL1L3	0.603262	0
430	GPR31	0.602777	0
431	CLECL1	0.602183	0
432	SPI1	0.601063	0
433	DOK3	0.60105	0
434	TNFRSF17	0.599721	0
435	SIGLEC1	0.599207	0
436	CR1	0.599041	0
437	LAP3	0.5989	0
438	AGAP2	0.598639	0
439	FCER1G	0.598581	0
440	SLC24A4	0.597453	0
441	CCL2	0.59733	0
442	TXK	0.597169	0
443	LST1	0.597076	0
444	TRIM69	0.595955	0
445	AIF1	0.595878	0
446	NLRP7	0.594702	0
447	APOL3	0.593927	0
448	CD1B	0.593656	0
449	C2	0.593539	0
450	CCL18	0.592118	0
451	LGALS9	0.591949	0
452	IFI16	0.591922	0
453	RAC2	0.591745	0
454	NMI	0.591368	0
455	TIMD4	0.591323	0
456	SLC9A9	0.591257	0
457	GIMAP7	0.591175	0
458	ST8SIA4	0.590788	0
459	CPVL	0.590734	0
460	IGLL5	0.590351	0
461	FLVCR2	0.589914	0
462	CASS4	0.589764	0
463	MX2	0.588477	0
464	RASGEF1B	0.587948	0
465	BATF2	0.587445	0
466	TLR10	0.58739	0
467	NLRC4	0.587165	0
468	CSF2RA	0.587152	0
469	IFI30	0.587069	0
470	CD79B	0.586819	0
471	SIGLEC7	0.586661	0
472	CCL19	0.585457	0
473	ACOD1	0.585427	0
474	PPP1R16B	0.584194	0
475	IFI44L	0.582387	0

476	CCL22	0.582129	0
477	NPL	0.581825	0
478	ARRDC5	0.581617	0
479	TNFRSF13I	0.581077	0
480	GTSF1	0.579994	0
481	PRDM1	0.579947	0
482	KLRC4-KLI	0.579529	0
483	IL1R2	0.578916	0
484	RUFY4	0.578734	0
485	CD163	0.578494	0
486	MILR1	0.578275	0
487	IFI44	0.578045	0
488	1-Mar	0.577669	0
489	CLEC10A	0.577381	0
490	CASP5	0.576843	0
491	SIRPB1	0.576343	0
492	HAPLN3	0.575504	0
493	HVCN1	0.574097	0
494	XAF1	0.572538	0
495	PILRA	0.572458	0
496	RNASE6	0.572135	0
497	SLFN14	0.570328	0
498	CELF2	0.570147	0
499	GBP6	0.568924	0
500	LILRA4	0.568867	0
501	CASP10	0.56752	0
502	SIRPB2	0.566976	0
503	CEACAM21	0.566488	0
504	HK3	0.566406	0
505	IL2	0.565771	0
506	KLHDC7B	0.565314	0
507	IFIT3	0.564621	0
508	PAG1	0.564231	0
509	SUCNR1	0.564113	0
510	HPSE	0.563995	0
511	PLEKHO2	0.562725	0
512	LY96	0.562575	0
513	FOXB1	0.562413	0
514	IFIH1	0.562138	0
515	DNAJC5B	0.561731	0
516	IL7	0.561376	0
517	NPFFR1	0.560675	0
518	CARMIL2	0.560317	0
519	DPEP2	0.560258	0
520	HSD11B1	0.560036	0
521	GLT1D1	0.559935	0
522	COL6A5	0.559319	0
523	GRAMD1B	0.55925	0
524	CCL17	0.558979	0
525	INSL3	0.557643	0
526	P2RX5	0.557416	0
527	UBASH3B	0.557339	0
528	CMKLR1	0.557129	0

529	RSAD2	0.556161	0
530	HLA-A	0.556081	0
531	NABP1	0.556005	0
532	APOBEC3C	0.554269	0
533	TMEM156	0.553621	0
534	NFAM1	0.553223	0
535	LGMN	0.552824	0
536	TMEM273	0.551885	0
537	ACSL5	0.55188	0
538	CAMK4	0.551567	0
539	COL4A4	0.550298	0
540	GPSM3	0.549843	0
541	PARP14	0.549663	0
542	SH2D1B	0.549079	0
543	CHST11	0.548223	0
544	HSF5	0.54781	0
545	DOCK8	0.547809	0
546	CCDC141	0.54763	0
547	IL18	0.547517	0
548	STK17B	0.547165	0
549	COTL1	0.547159	0
550	HLA-DRB5	0.54669	0
551	C3AR1	0.546481	0
552	FAM129C	0.546331	0
553	APOBEC3D	0.546241	0
554	CYSLTR2	0.546179	0
555	OASL	0.545194	0
556	FLI1	0.545005	0
557	DAZL	0.543263	0
558	ANKRD44	0.542802	0
559	RTP5	0.541463	0
560	STAMBPL1	0.541389	0
561	P2RX1	0.541263	0
562	CLEC2D	0.540806	0
563	RASSF2	0.539909	0
564	KLRG1	0.539575	0
565	TMEM140	0.539102	0
566	PPP1R18	0.53886	0
567	ZBTB32	0.538832	0
568	GIMAP2	0.538291	0
569	PTCRA	0.538177	0
570	P2RY14	0.537854	0
571	KIR3DL1	0.537217	0
572	STAT2	0.536638	0
573	EMILIN2	0.536338	0
574	CREB3L3	0.536015	0
575	OAS2	0.535293	0
576	ADGRE2	0.535172	0
577	RASSF5	0.534658	0
578	FCGR3A	0.534175	0
579	MRC1	0.533873	0
580	C5orf58	0.532358	0
581	CD300E	0.531486	0

582	STK17A	0.531466	0
583	CMPK2	0.531044	0
584	SPIC	0.530947	0
585	C1S	0.530549	0
586	CCRL2	0.529659	0
587	MSL3	0.529648	0
588	TSPAN32	0.528151	0
589	IFNAR2	0.527316	0
590	LRRC25	0.527012	0
591	FCER2	0.526937	0
592	ICAM1	0.526902	0
593	CR2	0.526869	0
594	ABI3	0.526726	0
595	PRDM8	0.526429	0
596	CCL4L2	0.526302	0
597	CD33	0.526265	0
598	TSHR	0.526062	0
599	PTPRO	0.52599	0
600	SLFN11	0.525831	0
601	CD1E	0.525748	0
602	UTS2	0.525648	0
603	CD101	0.525551	0
604	FAM20A	0.524435	0
605	PLSCR1	0.523833	0
606	CEACAM4	0.523764	0
607	TEX11	0.523256	0
608	RHOF	0.52251	0
609	LILRA6	0.522302	0
610	BCL2L14	0.520945	0
611	HERC5	0.520776	0
612	JAKMIP1	0.520576	0
613	DOCK11	0.520236	0
614	TOX2	0.519233	0
615	TNFRSF13(	0.518728	0
616	TYROBP	0.518069	0
617	KIR2DL3	0.518046	0
618	DUSP2	0.517202	0
619	SIGLEC14	0.516976	0
620	SOD2	0.516724	0
621	FCGR1A	0.516497	0
622	SLCO2B1	0.515673	0
623	RASGRP2	0.51473	0
624	DNAH8	0.514602	0
625	FYN	0.514552	0
626	TLR1	0.514536	0
627	IGF2BP3	0.514258	0
628	IL23R	0.513883	0
629	HMSD	0.512993	0
630	HLA-DQB2	0.512399	0
631	VPREB3	0.512342	0
632	PLCL2	0.51213	0
633	BTN2A2	0.511726	0
634	GPR84	0.511252	0

635	P2RX7	0.510921	0
636	ZNF804A	0.510699	0
637	GBP2	0.510403	0
638	FXYD2	0.510388	0
639	IGFLR1	0.510163	0
640	HS3ST3B1	0.510126	0
641	FUT4	0.509756	0
642	IL26	0.508897	0
643	NRROS	0.508857	0
644	GLIPR2	0.508534	0
645	MFNG	0.507491	0
646	KLRC4	0.507487	0
647	RAP2B	0.506709	0
648	ADAM19	0.506226	0
649	DPPA4	0.505771	0
650	GPR25	0.505691	0
651	ITGA4	0.5056	0
652	C3	0.50487	0
653	TRIM21	0.503965	0
654	TDRD6	0.50356	0
655	CCDC69	0.503406	0
656	RUBCNL	0.503062	0
657	MYH6	0.502827	0
658	DAPK1	0.502483	0
659	MIXL1	0.502448	0
660	MEP1A	0.502339	0
661	PTGDR	0.502059	0
662	PPP2R2B	0.501233	0
663	RGL4	0.501186	0
664	GIMAP6	0.500612	0
665	ETS1	0.499881	0
666	SLC1A3	0.49981	0
667	GPA33	0.499438	0
668	STK10	0.499397	0
669	PTGDS	0.499072	0
670	MEFV	0.498241	0
671	POU3F1	0.498222	0
672	ITPRIPL1	0.497939	0
673	CD300A	0.497688	0
674	ASGR2	0.49754	0
675	CLEC2B	0.497493	0
676	RNF19B	0.497425	0
677	SIRPD	0.496863	0
678	CFLAR	0.496755	0
679	ADCY7	0.495778	0
680	GPR150	0.495645	0
681	IL22RA2	0.495098	0
682	RTP4	0.494792	0
683	SH3BP1	0.494182	0
684	LAT2	0.493885	0
685	MYO7A	0.493656	0
686	FAS	0.493244	0
687	FCGR1B	0.492539	0

688	CSF1R	0.492283	0
689	PLEKHO1	0.491452	0
690	KIR2DL1	0.491168	0
691	CD1D	0.490919	0
692	ENTHD1	0.490471	0
693	CCR6	0.490396	0
694	FCGR2B	0.489619	0
695	KYNU	0.489502	0
696	TOGARAM2	0.489471	0
697	APOC1	0.489353	0
698	HLA-DQA2	0.489173	0
699	IFIT2	0.489131	0
700	BATF3	0.488567	0
701	SERPING1	0.488482	0
702	CASP4	0.48787	0
703	SNAI3	0.487846	0
704	VSIR	0.487624	0
705	BTN1A1	0.487464	0
706	PIK3R6	0.487444	0
707	SLC2A5	0.487376	0
708	FMNL2	0.487319	0
709	ADAP2	0.487219	0
710	EGFL6	0.485442	0
711	TLR4	0.485207	0
712	GIMAP1	0.485044	0
713	TLR6	0.484661	0
714	CD300C	0.484095	0
715	SHD	0.48383	0
716	PLA1A	0.483029	0
717	SIGLEC9	0.482209	0
718	FOXN2	0.482057	0
719	NLRP3	0.481821	0
720	LRRC8C	0.480707	0
721	DAPP1	0.480475	0
722	PDE3B	0.480373	0
723	ITIH1	0.480332	0
724	1-Sep	0.480195	0
725	JCHAIN	0.479458	0
726	FAM49A	0.47909	0
727	CXCR4	0.478841	0
728	RARRES1	0.478489	0
729	PSMB8	0.47787	0
730	CDHR1	0.477691	0
731	IFNL1	0.477446	0
732	TLR7	0.477225	0
733	CD209	0.477036	0
734	GP1BA	0.4765	0
735	ADA	0.47609	0
736	ARNTL2	0.475692	0
737	CLEC17A	0.47485	0
738	ECEL1	0.474206	0
739	AICDA	0.473732	0
740	GPR15	0.473651	0

741	6-Sep	0.472969	0
742	SH3KBP1	0.472785	0
743	CCL3	0.472722	0
744	LSP1	0.471798	0
745	ART3	0.471685	0
746	PSMB10	0.471594	0
747	CCL11	0.471265	0
748	TCN2	0.471166	0
749	STARD4	0.470633	0
750	APOL2	0.470133	0
751	PML	0.469715	0
752	C19orf38	0.469532	0
753	MCF2L2	0.469525	0
754	FNBP1	0.469411	0
755	LILRB5	0.468523	0
756	PARP12	0.468196	0
757	TCF7	0.467959	0
758	SAMD9	0.467645	0
759	SLFN5	0.467617	0
760	SLC2A6	0.467169	0
761	AOC1	0.466982	0
762	RAB42	0.466974	0
763	HERC6	0.466665	0
764	SLC8A1	0.466399	0
765	SYK	0.46637	0
766	OR52N4	0.466111	0
767	RAB39A	0.465309	0
768	OPTN	0.465208	0
769	ARL4C	0.465087	0
770	ITGAM	0.464863	0
771	SP110	0.464487	0
772	C1R	0.464203	0
773	ADAM28	0.463774	0
774	CSF2	0.463545	0
775	ARRB2	0.462459	0
776	ELMO1	0.462325	0
777	FCGR2A	0.461558	0
778	HEATR9	0.460668	0
779	HLA-C	0.460412	0
780	ITGAD	0.460363	0
781	TGM2	0.460262	0
782	CPNE5	0.460048	0
783	MX1	0.459816	0
784	ISL2	0.459252	0
785	ACSL4	0.459044	0
786	TLR2	0.459011	0
787	LPAR5	0.458309	0
788	UNC13D	0.458019	0
789	ANKRD33E	0.45788	0
790	GIMAP8	0.457832	0
791	FPR1	0.456959	0
792	CD1C	0.456702	0
793	DERL3	0.455581	0

794	C19orf84	0.455564	0
795	RASGRP3	0.455391	0
796	COL19A1	0.455336	0
797	MPP1	0.454659	0
798	RELT	0.454306	0
799	VNN1	0.453954	0
800	IFI27	0.453942	0
801	CTSL	0.453832	0
802	DGKG	0.453829	0
803	UBE2L6	0.453808	0
804	TNFRSF4	0.453043	0
805	KLHL34	0.452969	0
806	OSCAR	0.452938	0
807	CERKL	0.452576	0
808	SELE	0.452143	0
809	SOWAHD	0.45157	0
810	CHST2	0.451543	0
811	INTS6L	0.451082	0
812	PIM1	0.45095	0
813	GPR132	0.450769	0
814	FCHO1	0.44957	0
815	GRIP2	0.449042	0
816	MARCO	0.447891	0
817	SIRPA	0.447618	0
818	ADGRG3	0.447016	0
819	CCL23	0.44672	0
820	ARAP2	0.445345	0
821	VAMP5	0.444991	0
822	COL4A3	0.444469	0
823	TMEM176I	0.444414	0
824	ADGRE1	0.444066	0
825	RNF166	0.443695	0
826	ANKRD55	0.44325	0
827	MSN	0.443114	0
828	PIWIL4	0.443104	0
829	IL6R	0.443063	0
830	CGAS	0.442993	0
831	BTNL8	0.442975	0
832	WNT1	0.442921	0
833	SP100	0.442906	0
834	LY86	0.4425	0
835	CCDC88B	0.442057	0
836	CFP	0.44152	0
837	PMCH	0.440774	0
838	NR4A3	0.440099	0
839	EPHB2	0.439998	0
840	REC8	0.439663	0
841	KCNA2	0.438804	0
842	ZNF396	-0.43812	0
843	C5AR1	0.438066	0
844	TDO2	0.437478	0
845	NFATC2	0.437431	0
846	MOXD1	0.437378	0

847	RIN3	0.437176	0
848	AMPD1	0.437105	0
849	CLEC12B	0.436711	0
850	GNG2	0.436627	0
851	KLRF1	0.436448	0
852	GPBAR1	0.436438	0
853	CCL20	0.436326	0
854	TTC7A	0.436005	0
855	LBR	0.435801	0
856	RUNDC1	-0.43534	0
857	FCRL4	0.43527	0
858	LY75	0.434927	0
859	ANXA2R	0.434686	0
860	ST8SIA1	0.434604	0
861	CCND2	0.434569	0
862	GRIN3A	0.433645	0
863	RHEX	0.433529	0
864	LCNL1	0.432666	0
865	MYD88	0.432566	0
866	TNF	0.43217	0
867	STK4	0.432073	0
868	AQP9	0.432044	0
869	ANKRD22	0.431796	0
870	CARD6	0.431795	0
871	DHRS9	0.431743	0
872	FOXA1	-0.43164	0
873	CLEC1B	0.431642	0
874	CCL7	0.43152	0
875	GLIPR1	0.43121	0
876	FCAR	0.43114	0
877	EMP3	0.430903	0
878	P2RY13	0.430732	0
879	CYB5D2	-0.43057	0
880	TVP23A	0.430221	0
881	GPR141	0.429302	0
882	CSTA	0.429059	0
883	PSMA8	0.429006	0
884	GPNMB	0.428983	0
885	TCTN1	-0.42889	0
886	LTK	0.428676	0
887	EML4	0.427974	0
888	STAC3	0.427897	0
889	RASGRF1	0.427664	0
890	AKR1B1	0.427647	0
891	GM2A	0.427045	0
892	BHLHE22	0.426967	0
893	SEMA7A	0.426436	0
894	ARPC2	0.425622	0
895	DPYS	0.425504	0
896	TMEM71	0.42529	0
897	THSD4	-0.42524	0
898	TNFSF18	0.425202	0
899	KCNH4	0.424943	0

900	ACY3	0.424574	0
901	MATK	0.422152	0
902	MAN2B1	0.422143	0
903	TMC4	-0.42155	0
904	NT5DC4	0.421151	0
905	PNMA3	0.4209	0
906	PSAT1	0.420821	0
907	DOCK10	0.420479	0
908	ZEB2	0.419433	0
909	GMIP	0.419213	0
910	CDC42SE2	0.419182	0
911	TYMP	0.418816	0
912	TTC8	-0.41796	0
913	CYLD	0.417711	0
914	RFTN1	0.417097	0
915	TMEM52B	0.416762	0
916	TSPAN33	0.416293	0
917	ACTR3	0.415349	0
918	OAS3	0.414166	0
919	IGLL1	0.414146	0
920	NCOA7	0.414111	0
921	CD68	0.413839	0
922	ISG20	0.413187	0
923	S100A8	0.412983	0
924	S1PR2	0.412871	0
925	P4HTM	-0.41238	0
926	ZNF875	-0.41229	0
927	PLEKHA2	0.412286	0
928	SYT11	0.412054	0
929	LIPG	0.411904	0
930	PRAM1	0.411854	0
931	APOL4	0.411257	0
932	PLA2G12A	-0.41044	0
933	SUMF1	-0.41006	0
934	NFKBIE	0.409998	0
935	TRAPPC3L	0.4098	0
936	ADAM8	0.409689	0
937	C12orf74	0.409302	0
938	PLEKHG7	0.409302	0
939	ALOX5	0.409221	0
940	HHLA2	0.409142	0
941	TIGD6	-0.40887	0
942	PAQR8	0.408697	0
943	CCL25	0.408576	0
944	LRRC2	0.408554	0
945	OSM	0.408526	0
946	C1orf56	-0.40853	0
947	SHISAL2A	0.408315	0
948	NECAP2	0.408209	0
949	BCDIN3D	-0.40791	0
950	USP18	0.407429	0
951	SP140L	0.407188	0
952	CFH	0.406568	0

953	GATA3	-0.40629	0
954	LGI2	0.406283	0
955	PTGR2	-0.40619	0
956	NLRP1	0.40611	0
957	ARSG	-0.40593	0
958	C1orf54	0.405644	0
959	APOE	0.405254	0
960	PLA2G4A	0.404945	0
961	OLR1	0.404723	0
962	TOR4A	0.404596	0
963	CCDC82	0.404401	0
964	ESR2	0.404119	0
965	TLE4	0.404083	0
966	IL27	0.40402	0
967	PLTP	0.403826	0
968	SLC2A3	0.403552	0
969	SMTNL1	0.403474	0
970	ALOX5AP	0.403446	0
971	CLEC9A	0.402814	0
972	MFHAS1	0.402518	0
973	SMCO4	0.402475	0
974	GALNT12	0.402203	0
975	DDX60	0.402128	0
976	LACC1	0.401734	0
977	SH2B3	0.401642	0
978	CHIT1	0.401605	0
979	RPS6KA3	0.401535	0
980	ARHGAP4	0.401304	0
981	SPATC1	0.401237	0
982	ITM2C	0.401152	0
983	LHFPL2	0.401115	0
984	CACNA1E	0.401062	0
985	PTK2B	0.400948	0
986	IL23A	0.400424	0
987	LAT	0.400143	0
988	MZB1	0.400115	0
