# Supplementary material for: Molecular and clinical characterization of ICOS expression in breast cancer through large-scale transcriptome data
Source: PLoS One. 2023 Dec 21;18(12):e0293469. doi: 10.1371/journal.pone.0293469 (PMC10734928; doi:10.1371/journal.pone.0293469)
Supplement: S2 Table — (PDF) [file pone.0293469.s002.pdf]

Table S2. Genes significantly correlated with ICOS expression in the METABRIC cohort

	symbol	correlation	pvalue
1	P2RX1	0.407719	3.66E-77
2	IL21R	0.73377	1.24504542751994e-321
3	RASSF4	0.658085	1.11E-236
4	DOCK2	0.674497	6.12E-253
5	CD52	0.708239	6.41E-290
6	LRRC8C	0.439171	1.32E-90
7	CD40LG	0.53391	8.81E-141
8	ADCY7	0.423305	1.20E-83
9	CPVL	0.445463	1.81E-93
10	STX11	0.558612	1.08E-156
11	GLRB	-0.45323	4.36E-97
12	TCL1A	0.515338	1.17E-129
13	LY96	0.550355	3.17E-151
14	C14orf45	-0.43679	1.54E-89
15	WAS	0.692396	6.43E-272
16	REC8	0.433686	3.71E-88
17	EML4	0.530363	1.33E-138
18	ARHGAP9	0.755566	0
19	RAC2	0.682608	2.27E-261
20	NPL	0.555728	9.11E-155
21	SYK	0.559654	2.15E-157
22	IL2RA	0.620953	1.98E-203
23	LCK	0.644705	3.65E-224
24	SH3BP1	0.498259	5.05E-120
25	STAT2	0.442373	4.69E-92
26	TRV3J34	0.424285	4.59E-84
27	RNASE6	0.435357	6.73E-89
28	IRF8	0.715704	7.90E-299
29	TRANK1	0.401868	8.12E-75
30	APOBEC3H	0.763197	0
31	OAS2	0.414493	6.14E-80
32	HLA-DRA	0.560296	7.92E-158
33	TRAT1	0.721601	4.51E-306
34	DKFZp666(	0.529896	2.55E-138
35	SRGN	0.503681	5.05E-123
36	IDO1	0.791	0
37	XAF1	0.554053	1.18E-153
38	IFNAR2	0.434311	1.96E-88
39	IGLL3P	0.574444	1.28E-167
40	CD53	0.712186	1.36E-294
41	SLC9A9	0.535047	1.74E-141
42	RASAL3	0.72136	9.01E-306
43	RAI1	-0.40907	1.04E-77
44	CASC1	-0.4292	3.47E-86
45	FCHSD2	0.423909	6.65E-84
46	HLA-DMA	0.652481	2.32E-231
47	NUP62	0.618626	1.71E-201
48	DKFZp686(	0.627218	1.01E-208
49	TNFSF14	0.58428	1.03E-174
50	FYN	0.549178	1.85E-150
51	APOBEC3F	0.451731	2.21E-96

52	FCRL3	0.505326	6.06E-124
53	TNFRSF13I	0.616428	1.12E-199
54	NCOA7	0.426071	7.84E-85
55	DHRS9	0.410976	1.73E-78
56	IFNG	0.697729	7.59E-278
57	LST1	0.465715	4.15E-103
58	TNFRSF4	0.690303	1.26E-269
59	CRLF3	0.467629	4.72E-104
60	C2	0.557985	2.84E-156
61	PLCL2	0.553801	1.73E-153
62	TMEM30B	-0.40722	5.81E-77
63	ICAM2	0.41735	3.96E-81
64	MSN	0.457038	6.73E-99
65	APOE	0.532898	3.70E-140
66	RELB	0.446441	6.40E-94
67	RGS19	0.480389	1.65E-110
68	CD79B	0.710078	4.35E-292
69	MZB1	0.540433	7.43E-145
70	HLA-DQA1	0.577027	1.86E-169
71	FOXA1	-0.4401	5.03E-91
72	CHFR	0.419387	5.53E-82
73	GLIPR2	0.528138	2.99E-137
74	PSME2	0.523247	2.60E-134
75	RUNDC1	-0.47141	6.14E-106
76	CYBB	0.57074	5.22E-165
77	HUAT	0.514153	5.66E-129
78	LGALS9C	0.459467	4.59E-100
79	IL15	0.613705	1.88E-197
80	IL15RA	0.515086	1.63E-129
81	CAMK1G	0.599317	4.89E-186
82	KIAA0922	0.445522	1.70E-93
83	SAMD3	0.787376	0
84	BCL11B	0.709599	1.60E-291
85	CCL2	0.493762	1.42E-117
86	TRIM69	0.580385	7.12E-172
87	LTA	0.876913	0
88	HLA-E	0.679207	8.40E-258
89	HLA-DPB1	0.503836	4.14E-123
90	HAMP	0.425537	1.33E-84
91	GMIP	0.476835	1.10E-108
92	CDK14	-0.41471	4.97E-80
93	EFHD2	0.485116	5.70E-113
94	CD3G	0.837334	0
95	CXCL13	0.680806	1.79E-259
96	ITM2C	0.524452	4.95E-135
97	STC2	-0.41144	1.12E-78
98	APOBEC3G	0.709427	2.56E-291
99	HLA-DPA1	0.536545	2.04E-142
100	EMILIN2	0.456166	1.76E-98
101	KDM2B	0.426387	5.72E-85
102	LRRC48	-0.45847	1.38E-99
103	CCL13	0.577982	3.84E-170
104	CD27	0.799196	0

105	TNFRSF17	0.591321	6.11E-180
106	AIM2	0.810555	0
107	ADAM7	0.687016	4.54E-266
108	GZMK	0.768933	0
109	RFTN1	0.44808	1.12E-94
110	HCLS1	0.662746	3.41E-241
111	LGMN	0.40249	4.60E-75
112	CLEC12A	0.439944	5.92E-91
113	CCL21	0.416731	7.19E-81
114	TOX2	0.593515	1.35E-181
115	PMCH	0.466672	1.40E-103
116	ELF4	0.404068	1.08E-75
117	SAMSN1	0.538916	6.71E-144
118	FGL2	0.597319	1.70E-184
119	PTPRCAP	0.838395	0
120	BTN3A3	0.588374	9.77E-178
121	C1QC	0.569419	4.37E-164
122	HTRA4	0.477886	3.20E-109
123	RUNX3	0.760073	0
124	LOC64373[illegible]	0.514485	3.64E-129
125	RTP4	0.496193	6.80E-119
126	PRKX	0.490686	6.37E-116
127	SEL1L3	0.661904	2.26E-240
128	IFIT3	0.436358	2.41E-89
129	LRCH4	0.435621	5.14E-89
130	FLVCR2	0.613966	1.15E-197
131	GBP5	0.868244	0
132	MYO5A	0.413927	1.05E-79
133	DEF6	0.571205	2.47E-165
134	HCST	0.754342	0
135	KDM4B	-0.40992	4.68E-78
136	CD3D	0.871252	0
137	TGM2	0.471775	4.03E-106
138	AMICA1	0.699649	5.17E-280
139	FCRL5	0.537549	4.82E-143
140	ARHGAP15	0.665771	3.64E-244
141	MX1	0.468387	1.98E-104
142	CAPN8	-0.41041	2.95E-78
143	CMKLR1	0.52905	8.37E-138
144	TMEM71	0.450419	9.08E-96
145	DOCK10	0.477238	6.87E-109
146	GVIN1	0.726786	1.34452881435471e-312
147	IL1R2	0.4155	2.34E-80
148	RAB8B	0.416243	1.15E-80
149	MS4A6A	0.411596	9.61E-79
150	IGF2BP3	0.451744	2.17E-96
151	KLRD1	0.647815	5.12E-227
152	CD3E	0.779396	0
153	EHD1	0.456723	9.52E-99
154	LILRB4	0.595445	4.61E-183
155	PTGDS	0.532975	3.32E-140
156	TLR9	0.512982	2.68E-128
157	PITPNM2	0.504782	1.22E-123

158	DPEP2	0.54479	1.26E-147
159	APBB1IP	0.595589	3.58E-183
160	GZMB	0.798187	0
161	AIF1	0.402073	6.73E-75
162	SIRPA	0.427022	3.04E-85
163	GBP2	0.587422	4.99E-177
164	PSMB9	0.651563	1.68E-230
165	UBASH3B	0.541902	8.73E-146
166	P2RY10	0.630518	1.46E-211
167	CTSH	0.469163	8.16E-105
168	GBP4	0.799872	0
169	HLA-H	0.421491	7.13E-83
170	SOCS1	0.573931	2.96E-167
171	SLFN11	0.509922	1.52E-126
172	C5orf20	0.751572	0
173	IFI30	0.628209	1.43E-209
174	BX109404	-0.41046	2.80E-78
175	PSMB10	0.661232	1.02E-239
176	PILRA	0.415843	1.69E-80
177	CARD16	0.538978	6.13E-144
178	CD48	0.749239	0
179	PLCB2	0.544457	2.05E-147
180	ICOS	1	0
181	HLA-G	0.50543	5.29E-124
182	PIGV	-0.40784	3.28E-77
183	PAQR8	0.46165	4.03E-101
184	CDCA7	0.467948	3.28E-104
185	NAPSA	0.461734	3.67E-101
186	MLPH	-0.40227	5.63E-75
187	GBP1	0.713423	4.47E-296
188	IL18BP	0.793812	0
189	RHOF	0.446605	5.38E-94
190	CASP3	0.402986	2.92E-75
191	CCDC24	-0.42328	1.24E-83
192	PARP12	0.477626	4.35E-109
193	RARRES1	0.515472	9.74E-130
194	LPXN	0.728739	4.28493120915603e-315
195	PNOC	0.557254	8.76E-156
196	TYMP	0.481761	3.21E-111
197	FCRH3	0.74672	0
198	GZMH	0.646304	1.26E-225
199	ARRDC5	0.588059	1.68E-177
200	TYROBP	0.428796	5.18E-86
201	RASGRP3	0.541341	1.98E-145
202	NKG7	0.823591	0
203	BLM	0.416449	9.43E-81
204	IKBKE	0.404538	6.99E-76
205	LILRB3	0.617646	1.11E-200
206	PLEKHF1	0.445388	1.96E-93
207	ADAMDEC1	0.707451	5.38E-289
208	KLHL6	0.682087	8.04E-261
209	HLA-DOA	0.586062	5.06E-176
210	CCL22	0.571387	1.84E-165

211	PLCG2	0.631697	1.39E-212
212	TIGIT	0.562885	1.39E-159
213	PLAC8	0.649292	2.20E-228
214	TNFRSF1B	0.68365	1.79E-262
215	STK17B	0.472058	2.91E-106
216	C10orf125	0.441551	1.11E-91
217	CLEC4A	0.443491	1.45E-92
218	MAPK1	0.502555	2.14E-122
219	STAT1	0.743261	0
220	IFI44	0.503123	1.03E-122
221	C1orf54	0.490552	7.52E-116
222	TTC8	-0.43831	3.21E-90
223	IFIT2	0.467732	4.19E-104
224	MAPT	-0.44059	3.01E-91
225	VAMP5	0.504806	1.18E-123
226	CYB5D2	-0.46422	2.25E-102
227	TNF	0.47148	5.68E-106
228	TAP2	0.41471	4.99E-80
229	CD163	0.464278	2.11E-102
230	LAG3	0.743465	0
231	CYTIP	0.634033	1.27E-214
232	IGJ	0.407758	3.53E-77
233	WNT10A	0.608608	2.43E-193
234	POU2AF1	0.670146	1.58E-248
235	CD69	0.584541	6.66E-175
236	ELMO1	0.501872	5.13E-122
237	GNGT2	0.585765	8.38E-176
238	CD38	0.759542	0
239	PBX4	0.498275	4.95E-120
240	BTLA	0.692834	2.12E-272
241	LBH	0.403207	2.38E-75
242	PTGDR	0.43079	6.98E-87
243	AKNA	0.660216	9.84E-239
244	C14orf72	0.420692	1.55E-82
245	C13orf18	0.46323	6.86E-102
246	NPAS1	-0.40548	2.93E-76
247	VCAM1	0.609931	2.12E-194
248	PTGER4	0.543452	9.02E-147
249	C1QA	0.640572	2.01E-220
250	PHYHD1	-0.40389	1.27E-75
251	P2RY6	0.607939	8.32E-193
252	PLEKHA2	0.510755	5.09E-127
253	CCL4	0.548025	1.04E-149
254	LAT2	0.435149	8.33E-89
255	OXNAD1	0.441321	1.41E-91
256	CARD9	0.471671	4.55E-106
257	HK3	0.505132	7.78E-124
258	APOBEC3C	0.4284	7.70E-86
259	TCRVB	0.866001	0
260	PIM1	0.414538	5.88E-80
261	BTN3A1	0.660033	1.48E-238
262	INPP5D	0.64419	1.08E-223
263	NEK11	-0.41468	5.14E-80

264	GIMAP7	0.633915	1.61E-214
265	CD8A	0.813144	0
266	CSK	0.439763	7.15E-91
267	LY9	0.727788	7.09350568849691e-314
268	C14orf79	-0.40797	2.89E-77
269	NCF1C	0.677116	1.24E-255
270	LYL1	0.449225	3.28E-95
271	ARSG	-0.4108	2.04E-78
272	NAPSB	0.585075	2.70E-175
273	LAP3	0.499238	1.46E-120
274	E2F4	0.400524	2.76E-74
275	RSAD2	0.41478	4.67E-80
276	TNFAIP3	0.731087	3.98321652464963e-318
277	PIK3CD	0.710854	5.22E-293
278	BU935198	0.64183	1.48E-221
279	S1PR4	0.78394	0
280	MYO1G	0.707616	3.44E-289
281	IL18R1	0.612978	7.34E-197
282	GMFG	0.660977	1.80E-239
283	PTPN4	0.41804	2.04E-81
284	TEX11	0.473258	7.23E-107
285	PLA2G7	0.616733	6.26E-200
286	PLA1A	0.514	6.94E-129
287	RHBDF2	0.468544	1.66E-104
288	GZMA	0.79416	0
289	IL32	0.658191	8.80E-237
290	SH3KBP1	0.414048	9.38E-80
291	CTSC	0.577877	4.57E-170
292	TRAF3IP3	0.795732	0
293	PLEKHO1	0.629245	1.84E-210
294	NAGK	0.473852	3.62E-107
295	HLA-DOB	0.744931	0
296	SH2D2A	0.433845	3.16E-88
297	CCND2	0.409705	5.71E-78
298	DOCK8	0.542144	6.13E-146
299	TCRBV22S1	0.612374	2.27E-196
300	PTPN7	0.784886	0
301	LAT	0.657739	2.38E-236
302	PAG1	0.627172	1.10E-208
303	CELSR2	-0.46684	1.16E-103
304	CCR4	0.430712	7.55E-87
305	NR1H3	0.411459	1.09E-78
306	LYZ	0.497492	1.33E-119
307	DENND1C	0.629498	1.11E-210
308	FAM30A	0.566634	3.73E-162
309	NCR3	0.632092	6.30E-213
310	CD37	0.663577	5.24E-242
311	EMP3	0.409195	9.21E-78
312	ZNFX1	0.422359	3.05E-83
313	AA215505	0.475102	8.43E-108
314	UBASH3A	0.727093	5.46590995239696e-313
315	APOC1	0.531336	3.37E-139
316	KIAA0748	0.533065	2.93E-140

317	KLRC1	0.42855	6.63E-86
318	SPOCK2	0.838374	0
319	ABI3	0.585723	9.00E-176
320	CASP4	0.501563	7.61E-122
321	ARHGAP25	0.795873	0
322	FBXL2	-0.4121	5.98E-79
323	IGSF6	0.589885	7.30E-179
324	C11orf49	-0.40414	1.01E-75
325	FCER1G	0.42403	5.90E-84
326	KLHDC9	-0.42883	4.99E-86
327	IFI44L	0.510977	3.80E-127
328	IFI16	0.474808	1.19E-107
329	SOD2	0.517811	4.22E-131
330	ITGB2	0.587687	3.17E-177
331	CXCR5	0.710115	3.93E-292
332	IL7R	0.759817	0
333	E2F3	0.404245	9.16E-76
334	TCRBV4S1/	0.636892	3.85E-217
335	ZBED2	0.710522	1.30E-292
336	LILRA5	0.599606	2.93E-186
337	NCF1	0.52252	7.04E-134
338	CD79A	0.706767	3.39E-288
339	CD226	0.4907	6.26E-116
340	APH1B	-0.40398	1.17E-75
341	IFI27	0.427059	2.94E-85
342	TNFRSF25	0.44733	2.49E-94
343	ITGB7	0.731388	1.6173732982259e-318
344	PLEKHO2	0.54007	1.26E-144
345	BANK1	0.612307	2.57E-196
346	O1/O11 an	0.406546	1.09E-76
347	CSF2RA	0.485944	2.09E-113
348	RHOG	0.504579	1.59E-123
349	THEMIS	0.597995	5.12E-185
350	MAL	0.709871	7.64E-292
351	NMI	0.411518	1.03E-78
352	CD84	0.558452	1.38E-156
353	CYBA	0.485808	2.47E-113
354	GPR18	0.692646	3.41E-272
355	FCHO1	0.402475	4.66E-75
356	PASK	0.404762	5.69E-76
357	CTSL1	0.433195	6.11E-88
358	ANKRD22	0.472278	2.25E-106
359	PARP14	0.600521	5.71E-187
360	TNFAIP8	0.450352	9.76E-96
361	SEC14L1	0.400876	2.01E-74
362	FAM113B	0.485664	2.93E-113
363	CD72	0.70755	4.12E-289
364	SLC1A3	0.508507	9.70E-126
365	CD86	0.67376	3.46E-252
366	CD2	0.873367	0
367	CD8B	0.472379	2.00E-106
368	SLC15A3	0.582341	2.71E-173
369	CD70	0.424399	4.10E-84

370	CTSZ	0.439296	1.16E-90
371	ST8SIA4	0.509782	1.83E-126
372	PATL2	0.654851	1.35E-233
373	SLA2	0.725793	2.45306807818659e-311
374	STK4	0.624765	1.23E-206
375	TSPAN33	0.53194	1.44E-139
376	OBFC2A	0.56608	8.99E-162
377	TARP	0.463342	6.05E-102
378	RNF166	0.417182	4.66E-81
379	DRAM1	0.470203	2.47E-105
380	SLAMF7	0.564993	5.02E-161
381	CXCR3	0.840541	0
382	TRPV2	0.679758	2.23E-258
383	ARHGAP30	0.755169	0
384	STAG3	0.423044	1.56E-83
385	1-Sep	0.422756	2.07E-83
386	TLR10	0.466929	1.05E-103
387	KIF21B	0.545823	2.73E-148
388	IL10RA	0.746144	0
389	ITGAX	0.522036	1.36E-133
390	GPR183	0.552366	1.52E-152
391	ZMYND10	-0.41647	9.26E-81
392	CXCL11	0.440302	4.08E-91
393	CD74	0.443785	1.06E-92
394	LOC43994[illegible]	0.632026	7.18E-213
395	POP5	-0.46931	6.93E-105
396	MYD88	0.435087	8.88E-89
397	FCAR	0.595381	5.15E-183
398	IFIH1	0.529647	3.63E-138
399	FASLG	0.591168	7.96E-180
400	TRAF1	0.634021	1.30E-214
401	MAP4K1	0.8031	0
402	MEIS3	-0.43539	6.51E-89
403	BTN3A2	0.544514	1.89E-147
404	MEI1	0.58711	8.49E-177
405	C17orf97	-0.43006	1.46E-86
406	C6orf97	-0.40255	4.36E-75
407	VPREB3	0.607596	1.56E-192
408	LOC90925	0.43907	1.47E-90
409	PRF1	0.770407	0
410	CARD11	0.508938	5.52E-126
411	TMEM140	0.59396	6.21E-182
412	SASH3	0.79219	0
413	BATF2	0.503114	1.05E-122
414	IGLL1	0.630554	1.36E-211
415	CDC25B	0.411297	1.27E-78
416	CD300A	0.424414	4.04E-84
417	ZAP70	0.794022	0
418	PLEK	0.744358	0
419	LCP2	0.578294	2.29E-170
420	IL12RB1	0.476279	2.12E-108
421	CST7	0.773616	0
422	SLC7A7	0.575564	2.05E-168

423	TIMD4	0.455704	2.91E-98
424	PSAT1	0.474969	9.84E-108
425	WARS	0.699927	2.50E-280
426	DDX39A	0.416681	7.55E-81
427	KLRG1	0.603765	1.67E-189
428	MAFF	0.583727	2.63E-174
429	SEMA4D	0.712011	2.19E-294
430	SALL2	-0.4034	1.99E-75
431	GIMAP4	0.696159	4.35E-276
432	B2M	0.437194	1.02E-89
433	CD28	0.477227	6.97E-109
434	HAPLN3	0.636171	1.67E-216
435	C2orf88	0.432087	1.88E-87
436	TNFSF13B	0.691822	2.74E-271
437	HLA-DMB	0.705173	2.42E-286
438	C11orf75	0.406481	1.16E-76
439	CD83	0.568659	1.48E-163
440	RLTPR	0.463011	8.78E-102
441	CD5	0.755896	0
442	TBC1D10C	0.824746	0
443	IL16	0.445384	1.97E-93
444	PRKCB	0.789984	0
445	BIN1	0.414435	6.49E-80
446	PDE3B	0.411604	9.54E-79
447	HLA-F	0.728583	6.78952931375475e-315
448	P4HTM	-0.46236	1.82E-101
449	LRRC33	0.483362	4.72E-112
450	FLNB	-0.40787	3.19E-77
451	STK10	0.592232	1.26E-180
452	FNBP1	0.526561	2.68E-136
453	GRAP	0.499649	8.70E-121
454	GAMT	-0.41441	6.64E-80
455	LTB	0.840207	0
456	NUP93	0.436674	1.74E-89
457	HLA-B	0.613816	1.53E-197
458	LYAR	0.488274	1.23E-114
459	FPR3	0.61257	1.57E-196
460	AK056817	0.538327	1.57E-143
461	IPCEF1	0.45045	8.79E-96
462	GNLY	0.758969	0
463	GIMAP5	0.713439	4.27E-296
464	TAGAP	0.529641	3.65E-138
465	LAPTM5	0.562499	2.54E-159
466	RASSF2	0.576132	8.09E-169
467	CORO1A	0.765044	0
468	CD80	0.474533	1.64E-107
469	CCR7	0.816716	0
470	CELF2	0.511192	2.86E-127
471	MCOLN2	0.71173	4.74E-294
472	PPP1R16B	0.704408	1.86E-285
473	MX2	0.552989	5.92E-153
474	ICAM3	0.501462	8.66E-122
475	BBS1	-0.46976	4.10E-105

476	GPBAR1	0.429844	1.81E-86
477	CXCL9	0.855944	0
478	TCN2	0.495142	2.53E-118
479	NLRP7	0.403511	1.80E-75
480	IGFLR1	0.646226	1.49E-225
481	IFT88	-0.40656	1.08E-76
482	TNFRSF9	0.564346	1.40E-160
483	MATK	0.576651	3.45E-169
484	TCTN1	-0.45161	2.52E-96
485	DAPK1	0.478249	2.09E-109
486	WIPF1	0.499684	8.32E-121
487	EPSTI1	0.662512	5.77E-241
488	MLKL	0.470559	1.64E-105
489	HSD11B1	0.460353	1.71E-100
490	SLAMF1	0.804553	0
491	RASSF5	0.587808	2.58E-177
492	SPI1	0.531576	2.40E-139
493	MYO1F	0.574733	8.00E-168
494	TDO2	0.446873	4.05E-94
495	RENBP	0.490817	5.42E-116
496	NFE2L3	0.466012	2.97E-103
497	CDK12	0.526546	2.74E-136
498	FKBP11	0.423865	6.94E-84
499	SLA	0.654383	3.74E-233
500	FAM129C	0.431381	3.84E-87
501	GPR132	0.570447	8.36E-165
502	C17orf87	0.654339	4.12E-233
503	BATF3	0.58033	7.81E-172
504	MGAT1	0.48563	3.06E-113
505	C1QB	0.587577	3.82E-177
506	SH2D1A	0.801496	0
507	GATA3	-0.43159	3.10E-87
508	PIP4K2A	0.50924	3.72E-126
509	CCR2	0.536114	3.79E-142
510	RCSD1	0.595558	3.78E-183
511	RSPH1	-0.43645	2.19E-89
512	PPP2R2B	0.49337	2.31E-117
513	FOXN2	0.582794	1.27E-173
514	FERMT3	0.752093	0
515	S100A8	0.421622	6.27E-83
516	C4orf7	0.405248	3.63E-76
517	RGS18	0.532221	9.66E-140
518	NLRC5	0.64801	3.39E-227
519	PTPRC	0.559733	1.90E-157
520	SLC2A6	0.536783	1.45E-142
521	SERPINA11	-0.4068	8.61E-77
522	LAIR2	0.666464	7.49E-245
523	KLRB1	0.653793	1.35E-232
524	PIM2	0.652054	5.85E-231
525	PARVG	0.632853	1.37E-213
526	PRDM1	0.474889	1.08E-107
527	DOK2	0.561035	2.51E-158
528	IRF1	0.732603	4.21932061548425e-320

529	LZTFL1	-0.43145	3.58E-87
530	FAM78A	0.430067	1.45E-86
531	UNC13D	0.46894	1.05E-104
532	6-Sep	0.632856	1.36E-213
533	RAPGEF1	0.430478	9.56E-87
534	ZNF831	0.765851	0
535	CD1B	0.50198	4.47E-122
536	RAB33A	0.554682	4.51E-154
537	CXCL10	0.75178	0
538	LGALS2	0.519036	8.06E-132
539	PDCD1	0.712772	2.70E-295
540	SH3BGRL3	0.439023	1.54E-90
541	CCL17	0.450927	5.25E-96
542	CD247	0.882392	0
543	MAN2B1	0.460018	2.49E-100
544	CD7	0.843095	0
545	ISG20	0.602597	1.38E-188
546	BLK	0.525157	1.87E-135
547	GIMAP6	0.487215	4.47E-114
548	CTRL	0.444754	3.83E-93
549	LCP1	0.712154	1.48E-294
550	CLECL1	0.52235	8.88E-134
551	KBTBD8	0.605997	2.90E-191
552	ETV7	0.52871	1.34E-137
553	HMHA1	0.428144	9.94E-86
554	BTK	0.468741	1.32E-104
555	C16orf61	0.453007	5.53E-97
556	CTLA4	0.874131	0
557	PTPN6	0.488447	9.93E-115
558	PVRIG	0.821956	0
559	IGF1R	-0.43502	9.53E-89
560	SP140	0.793207	0
561	GPSM3	0.59299	3.37E-181
562	LIME1	0.653142	5.55E-232
563	CD40	0.638956	5.61E-219
564	UBD	0.686343	2.40E-265
565	abParts	0.571985	6.99E-166
566	IL2RG	0.689985	2.78E-269
567	TLR8	0.626174	7.82E-208
568	NCF4	0.623414	1.71E-205
569	WDR19	-0.41624	1.15E-80
570	IL18RAP	0.759131	0
571	CXCR6	0.803638	0
572	TRIM21	0.531244	3.84E-139
573	SUCNR1	0.418296	1.59E-81
574	EFHC1	-0.44081	2.40E-91
575	RASGRP2	0.516051	4.49E-130
576	PTPN22	0.50873	7.25E-126
577	CTSW	0.695049	7.51E-275
578	SLCO2B1	0.427866	1.31E-85
579	BCL11A	0.524417	5.20E-135
580	RNF126P1	0.507596	3.18E-125
581	CD6	0.840891	0

582	FAM26F	0.478864	1.01E-109
583	CCL8	0.599329	4.79E-186
584	SPIB	0.595126	8.06E-183
585	TMEM176E	0.431315	4.11E-87
586	FCN1	0.644185	1.09E-223
587	FGR	0.619419	3.77E-202
588	BG205162	0.520354	1.35E-132
589	CDC42SE2	0.408801	1.33E-77
590	CCDC103	-0.41325	2.00E-79
591	EAF2	0.491899	1.43E-116
592	CD4	0.656417	4.38E-235
593	TCRA	0.697413	1.72E-277
594	CYTH1	0.545493	4.46E-148
595	ADAM19	0.422209	3.53E-83
596	GAB3	0.541857	9.33E-146
597	SELL	0.745473	0
598	THSD4	-0.41113	1.49E-78
599	CD97	0.688451	1.28E-267
600	DUSP2	0.453529	3.13E-97
601	IRF4	0.460472	1.50E-100
602	RGL4	0.749607	0
603	VAV1	0.544024	3.89E-147
604	KYNU	0.443919	9.23E-93
605	ACSL5	0.507146	5.72E-125
606	PRDM8	0.439457	9.83E-91
607	EVI2B	0.641828	1.49E-221
608	CASP1	0.602534	1.54E-188
609	PCNX	0.429147	3.64E-86
610	C1orf162	0.62172	4.53E-204
611	CR603183	0.522872	4.34E-134
612	BX099468	0.407989	2.84E-77
613	P2RY8	0.772232	0
614	CCL19	0.64065	1.71E-220
615	STAMBPL1	0.550155	4.27E-151
616	LRMP	0.646072	2.05E-225
617	ST3GAL2	0.430114	1.38E-86
618	ARPC2	0.444146	7.27E-93
619	ARHGAP4	0.59827	3.15E-185
620	AZGP1	-0.47425	2.27E-107
621	ITGA4	0.406391	1.26E-76
622	CD19	0.713574	2.94E-296
623	BIRC3	0.562964	1.23E-159
624	ADA	0.58712	8.34E-177
625	ACAP1	0.815732	0
626	FGD2	0.666358	9.55E-245
627	GHRL	0.403406	1.98E-75
628	IQCK	-0.41081	2.03E-78
629	CCR1	0.483048	6.88E-112
630	STAT4	0.807178	0
631	EOMES	0.738303	0
632	LAMP3	0.82793	0
633	LYN	0.601597	8.30E-188
634	PSTPIP1	0.724298	0.00E+00

635	TAP1	0.743377	0
636	C5orf39	0.603217	4.49E-189
637	PYHIN1	0.822662	0
638	EPHB6	0.421146	9.99E-83
639	CCM2	0.44639	6.76E-94
640	NAPOR-1	0.654468	3.11E-233
641	TIFAB	0.473029	9.43E-107
642	DKFZp761I	-0.40798	2.88E-77
643	TOX	0.421301	8.58E-83
644	KIAA1949	0.59179	2.71E-180
645	CIRBP	-0.40333	2.12E-75
646	ZBTB32	0.532355	7.99E-140
647	SLC2A5	0.444642	4.31E-93
648	ITK	0.867074	0
649	GPR65	0.433998	2.70E-88
650	CCL5	0.851488	0
651	NFS1	0.820866	0
652	NPSR1	0.590727	1.71E-179
653	UPP1	0.464886	1.06E-102
654	GRK6	0.436968	1.29E-89
655	RRNAD1	-0.44374	1.12E-92
656	DOCK11	0.531741	1.90E-139
657	OASL	0.563176	8.79E-160
658	SLC39A6	-0.40114	1.58E-74
659	ZNF683	0.749667	0
660	NCKAP1L	0.68124	6.27E-260
